# *PTCH1* and *CTNNB1* emerge as pivotal predictors of resistance to neoadjuvant chemotherapy in ER+/HER2- breast cancer

**DOI:** 10.3389/fonc.2023.1216438

**Published:** 2023-08-28

**Authors:** Gulnihal Ozcan

**Affiliations:** ^1^ Department of Medical Pharmacology, Koç University School of Medicine, Istanbul, Türkiye; ^2^ Koç University Research Center for Translational Medicine (KUTTAM), Istanbul, Türkiye

**Keywords:** breast cancer, chemoresistance, taxane-based neoadjuvant chemotherapy, predictive markers, precision medicine, bioinformatics

## Abstract

**Introduction:**

Endeavors in the molecular characterization of breast cancer opened the doors to endocrine therapies in ER+/HER2- breast cancer, increasing response rates substantially. Despite that, taxane-based neoadjuvant chemotherapy is still a cornerstone for achieving breast-conserving surgery and complete tumor resection in locally advanced cancers with high recurrence risk. Nonetheless, the rate of chemoresistance is high, and deselecting patients who will not benefit from chemotherapy is a significant task to prevent futile toxicities. Several multigene assays are being used to guide decisions on chemotherapy. However, their development as prognostic assays but not predictive assays limits predictive strength, leading to discordant results. Moreover, high costs impediment their use in developing countries. For global health equity, robust predictors that can be cost-effectively incorporated into routine clinical management are essential.

**Methods:**

In this study, we comprehensively analyzed 5 GEO datasets, 2 validation sets, and The Cancer Genome Atlas breast cancer data to identify predictors of resistance to taxane-based neoadjuvant therapy in ER+/HER2- breast cancer using efficient bioinformatics algorithms.

**Results:**

Gene expression and gene set enrichment analysis of 5 GEO datasets revealed the upregulation of 63 genes and the enrichment of CTNNB1-related oncogenic signatures in non-responsive patients. We validated the upregulation and predictive strength of 18 genes associated with resistance in the validation cohort, all exhibiting higher predictive powers for residual disease and higher specificities for ER+/HER2- breast cancers compared to one of the benchmark multi-gene assays. Cox Proportional Hazards Regression in three different treatment arms (neoadjuvant chemotherapy, endocrine therapy, and no systemic treatment) in a second comprehensive validation cohort strengthened the significance of *PTCH1* and *CTNNB1* as key predictors, with hazard ratios over 1.5, and 1.6 respectively in the univariate and multivariate models.

**Discussion:**

Our results strongly suggest that PTCH1 and CTNNB1 can be used as robust and cost-effective predictors in developing countries to guide decisions on chemotherapy in ER +/HER2- breast cancer patients with a high risk of recurrence. The dual function of PTCH1 as a multidrug efflux pump and a hedgehog receptor, and the active involvement of CTNNB1 in breast cancer strongly indicate that *PTCH1* and *CTNNB1* can be potential drug targets to overcome chemoresistance in ER +/HER2- breast cancer patients.

## 1 Introduction

Breast Cancer is the most frequent cancer and the leading cause of mortality from cancer in women worldwide (1). Elaborate investigation of the molecular mechanisms revealed the significance of estrogen receptor (ER), progesterone receptor (PR), and human epidermal growth factor receptor (HER2) in breast cancer. This knowledge enabled the classification of breast cancer into three subtypes; ER+/HER2-, HER2+, and triple-negative breast cancer (TNBC) which lacks all three receptors.

ER+/HER2- breast cancers constitute more than 70% of breast cancer cases, mainly consisting of luminal A and luminal B PAM50 intrinsic subtypes (2). Luminal A-type breast cancer is characterized by ER positivity, HER2 negativity, high expression of PR, and low Ki67. Luminal B-type breast cancers are also ER+ cancers but HER2 status may be negative or positive, PR expression may be low, and Ki67 may be high, in contrast to the luminal A-type (3, 4).

The mainstay of systemic therapy in ER+/HER2- breast cancers is endocrine therapy. However, resistance to endocrine therapy is a handicap in locally advanced breast cancer patients with high risk, leading to inadmissible recurrence rates (5). In this patient group taxane-based neoadjuvant chemotherapy is crucial to prevent relapse, especially in luminal B-type ER+/HER2- breast cancers. Neoadjuvant chemotherapy is crucial for down-staging the tumors to achieve complete tumor resection and breast-conserving surgery in locally advanced breast cancer patients with high recurrence risk. Moreover, neoadjuvant chemotherapy may provide a chance to guide decisions on adjuvant chemotherapy based on the response to neoadjuvant therapy (3, 5, 6). Nonetheless, response rates to taxane-based neoadjuvant chemotherapy are low in ER+/HER2- breast cancer patients compared to HER2+ breast cancers and TNBC (4, 6, 7). Since chemotherapeutics come with a cost of non-specific toxicities to normal tissues, deselecting patients who will not benefit from neoadjuvant chemotherapy is crucial to refrain from the unnecessary toxicities of chemotherapeutics.

The last two decades had witnessed intensive efforts to develop multi-gene assays for guiding decisions on therapy for breast cancer patients. Among several of these, Oncotype DX, MammaPrint, Endopredict, Prosigna, and Breast Cancer Index are incorporated into treatment guidelines as tools that may be used in patients where decisions on systemic chemotherapy are indefinite after primary clinical assessment (4). However, these multi-gene expression assays were originally developed as prognostic assays to estimate the risk of recurrence after endocrine therapy, but not to predict whether high-risk patients will respond to chemotherapy. Later trials on their predictive utility proposed these tests as tools that can provide insight for decisions on systemic chemotherapy. Despite that, the risk scores predicted by different multi-gene assays are commonly discordant and the benefits they provide over the 4-gene IHC assay (IHC4), which involves immunohistochemical analysis of the ER, PR, HER2, and the proliferation marker Ki67, is unclear. For instance, one of the most used benchmark assays, Oncotype Dx, consists of two main gene groups: ER-related genes and Ki67-related genes. If the expression of ER-related genes is high, the patient is considered low risk and undergoes endocrine therapy. On the other hand, patients with a high expression of Ki67-related genes are considered high risk and undergo neoadjuvant chemotherapy (8–12).

Another obstacle to the use of these multi-gene assays is their costs. Although, countries in which public health insurance systems reimburse these tests, like the United Kingdom and Germany, got benefit in the prediction of patients who will not respond to therapy (13, 14), limited coverage of health insurance systems in many developing countries impediment the chance of incorporating these tests into routine clinical management (15). For global health equity, the identification of robust predictors of resistance that can be cost-effectively incorporated into clinical management is required in breast cancer. Such predictive markers may also provide a chance for the selection of patients eligible for newly developed molecular targeted agents in the first-line setting, before subjecting them to the effects of chemotherapeutics.

**Figure 1:**
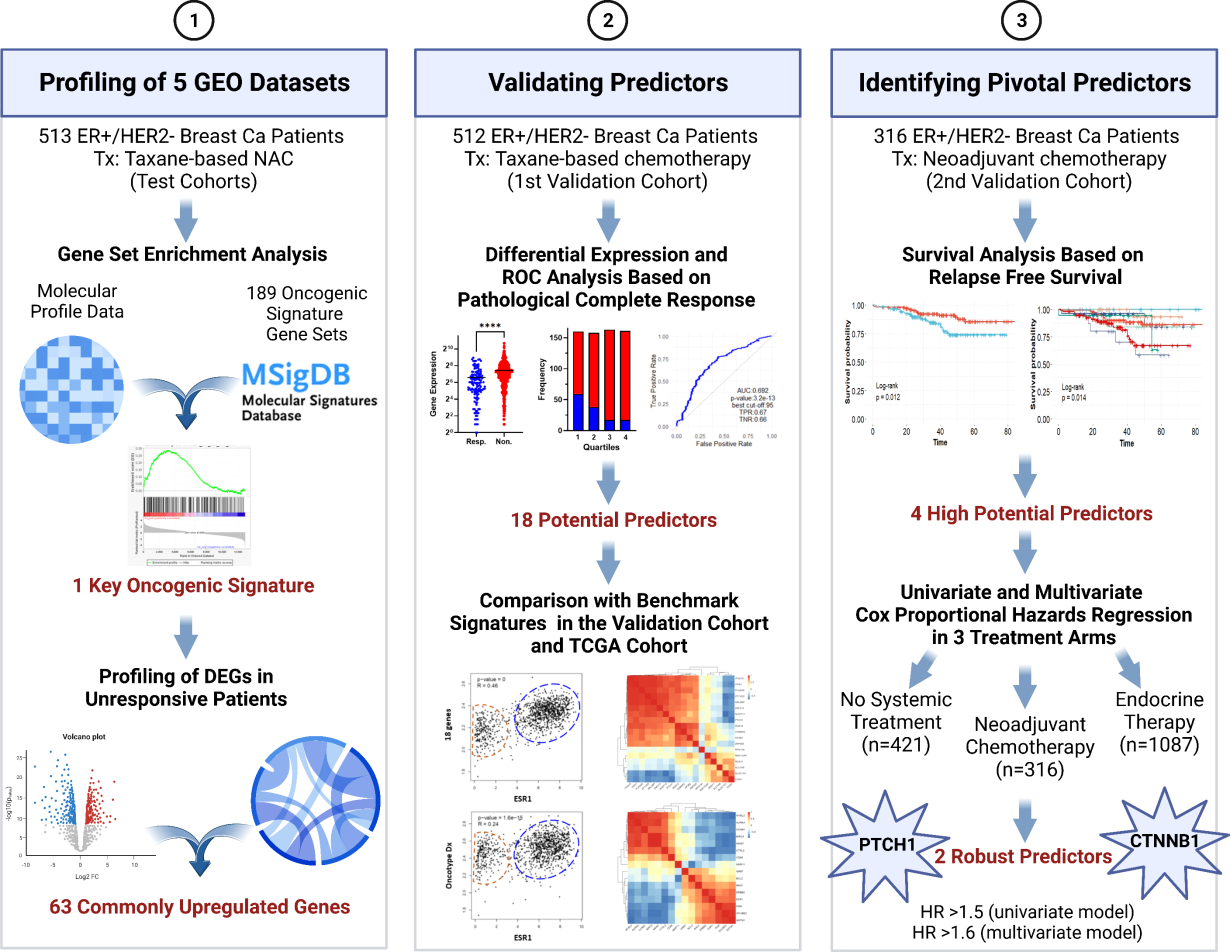
The algorithm used in the study for identifying pivotal predictors in ER+/HER2- breast cancer. DEG, differentially expressed genes; NAC, neoadjuvant chemotherapy; ROC, Receiver/Relative-operating characteristics; Tx, treatment. Created with BioRender.com.

In this study, we aimed to identify pivotal predictors for resistance to taxane-based neoadjuvant therapy in ER+/HER2- breast cancer that would guide decisions on neoadjuvant chemotherapy. To this end, we analyzed five GEO breast cancer datasets including a total of 513 patients. We identified the enrichment of β-catenin (CTNNB1)-related oncogenic signatures and 63 commonly upregulated genes associated with resistance. For validation of the upregulation and predictive strength of these 63 genes, we utilized a cohort of 512 ER+/HER2- patients who had undergone taxane-based systemic therapy in the ROC plotter database developed by Fekete & Győrffy for the validation of predictive markers in cancer (16). We validated that, 18 genes out of 63 upregulated genes had high and significant predictive values for residual disease in ER+/HER2- breast cancer. We comparatively analyzed these 18 genes with the most used multi-gene assay signatures in this validation cohort and The Cancer Genome Atlas (TCGA) dataset. With further analysis in a second cohort of 316 ER+/HER2- breast cancer patients who underwent neoadjuvant therapy in the KM plotter database by Győrffy et al. (17, 18), we validated the significance of 4 out of 18 genes together with CTNNB1 in relapse-free survival. Lastly, Cox Proportional Hazards Regression put forward PTCH1 and CTNNB1 as key markers of resistance to neoadjuvant therapy in ER+/HER2- breast cancer. **Figure 1** summarizes the algorithms we used to identify these 2 robust predictors.

## 2 Materials and methods

### 2.1 Data collection and identification of differentially expressed genes

To investigate the markers of resistance to taxane-based neoadjuvant chemotherapy in ER+/HER2- breast cancer, we analyzed GSE20194, GSE20271, GSE25055, GSE25065, and GSE32646 datasets in GEO (https://www.ncbi.nlm.nih.gov/geo/). All datasets included mRNA-sequencing data from fine needle aspiration biopsy (FNA) or core biopsy (CBX) samples collected from patients before surgery and any systemic therapy (19–25). Only patients with ER-positivity and HER2-negativity were included in the analysis. We did not include patients with other receptor subtypes of breast cancer, patients who did not receive taxane-based neoadjuvant therapy, or for whom information on the chemotherapy and the response to therapy was not available. Pathological complete response (pCR) was accepted as the surrogate of sensitivity to chemotherapy and residual disease (RD) was accepted as the surrogate of chemoresistance. Information on the number of patients with RD or pCR, chemotherapy regimens, and PAM50 intrinsic subtypes is summarized in **Supplementary Table 1**.

To identify differentially expressed genes (DEGs) in chemoresistant patients compared with chemosensitive patients, we used the GEO2R web tool (https://www.ncbi.nlm.nih.gov/geo/geo2r/). In total, we analyzed samples from 468 chemoresistant and 45 chemosensitive patients with ER+/HER2- breast cancer. Since response rates to taxane-based chemotherapy are low in ER+/HER2- breast cancer, the number of chemosensitive patients was much lower compared to the number of chemoresistant patients in all datasets. To avoid bias that could be caused by the imbalance in the number of resistant vs. sensitive patients or the inhomogeneous distribution of data, we applied log transformation and force normalization to all datasets. The p-value cut-off was selected as 0.05 for statistical significance. Genes with a log-fold change smaller than -0.2 were accepted as downregulated genes and genes with a log-fold change greater than 0.2 were accepted as upregulated genes. The volcano plots for DEGs were plotted on Image GP (http://www.ehbio.com/ImageGP). To identify the DEGs and ontologies shared by different datasets we analyzed the data on Metascape (26) (https://metascape.org) and extracted the circos plots.

### 2.2 Gene set enrichment analysis

To dissect the enriched hallmark gene sets and oncogenic signature gene sets in ER+/HER2- breast tumors resistant to taxane-based neoadjuvant chemotherapy, we performed gene set enrichment analysis on Gene Set Enrichment Analysis software GSEA_4.2.3 (27). First, we prepared the list of t values calculated in GEO2R for the differential expression of each gene in non-responsive patients compared to the responsive patients in each dataset. Then we uploaded the pre-ranked t-value lists for each dataset separately to the GSEA_4.2.3. We have chosen hallmark gene sets (50 sets) or oncogenic signature gene sets (189 sets) from Molecular Signatures Database (MSigDB) and ran the GSEA-Preranked tool (28, 29). We evaluated the GSEA plots, enrichment scores, normalized enrichment scores, and p-values for each reference gene set to find out statistically enriched hallmark genes and oncogenic signatures in 5 GEO datasets.

### 2.3 Functional annotation, enrichment, and hierarchical clustering analysis

To identify the gene ontologies and pathways that the DEGs were enriched, we analyzed the list of 63 commonly upregulated genes in non-responsive patients on The Database for Annotation, Visualization, and Integrated Discovery (DAVID) (Version 6.8) (https://david.ncifcrf.gov/). The gene ontologies (GO-CC: cellular compartments, GO-MF: molecular functions, and GO-BP: biological processes) and Kyoto Encyclopedia of Genes and Genomes (KEGG) pathways listed in the top enrichment clusters were explored (p-value significance cut-off: 0.05).

### 2.4 Gene expression profiling and receiver operating characteristic analysis

To validate the upregulation of key genes in patients who did not respond to taxane-based therapy, and demonstrate their specificity to ER+/HER2- breast cancer, we analyzed the data for 512 ER+/HER2- patients (437 non-responders vs 75 responders), 71 HER2+/ER- patients (31 non-responders vs. 40 responders), 204 TNBC patients (125 non-responders vs. 79 responders) who received taxane-based chemotherapy on the ROC-plotter database (16). The patients who received endocrine therapy or anti-HER2 therapy were not included in the gene expression profiling and ROC curve analysis. Pathological complete response was considered as the surrogate for responsiveness in both analysis types.

We evaluated the fold-change in gene expression in non-responders vs. responders and p-values calculated with the Mann-Whitney test (p-value cut-off=0.05). We also evaluated the frequency of responders and non-responders at each quartile of gene expression. The graphs for this analysis were plotted in GraphPad Prism 9. To validate the value of the key markers in predicting resistance to taxane-based chemotherapy, we analyzed the Receiver/Relative Operating Characteristic (ROC) curves of the genes in the ROCplotter (16). The data for the true positive rate (TPR), and true negative rate (TNR) calculated by the “pROC” package in R was used to plot ROC curves with the “ggplot2” package in R. We evaluated the area under the curve (AUC), ROC p-values, and calculated the positive predictive values (PPV) and negative predictive values (NPV) using the TPR, and TNR values extracted from the ROC analysis.

### 2.5 Kaplan Meier survival analysis and Cox proportional hazards regression

To investigate the effect of upregulated genes on survival we analyzed the KM-survival for 316 ER+/HER2- breast cancer patients who underwent neoadjuvant chemotherapy on the Kaplan-Meier Plotter database (17). For all genes, we downloaded the gene expression data (both categorical and continuous data) and relapse-free survival data for these 316 patients to perform KM survival analysis and Cox Proportional Hazards Regression. We built the univariate and multivariate survival models and Cox Proportional Hazards models with these genes using the ‘survival’ package in R. In KM analysis we included categorical expression of genes as high or low. We plotted the KM-survival plots using the ‘survminer’ and ‘ggplot2’ packages in R. We extracted the log-rank p-values for each model. The Proportional Hazards assumption for Cox models was tested with the Schoenfeld test in R using the ‘survival’ and ‘survminer’ packages in R. The FDR-adjusted p-values for Cox Proportional Hazards models were calculated by the ‘p.adjust’ package in R using the “Benjamini-Hochberg” method.

To validate the potential of *PTCH1*, and *CTNNB1* as predictors in ER+/HER2- breast cancer patients, we additionally extracted data for 421 ER+/HER2- patients who had not undergone any systemic therapy and 1087 ER+/HER2- patients who underwent endocrine therapy in KM plotter database. We established Cox Proportional Hazards models using *PTCH1*, and *CTNNB1* as covariates in three treatment arms: no systemic therapy, neoadjuvant chemotherapy, and endocrine therapy. To build Cox models, we used the ‘survival’ package in R and included gene expression values as log2-normalized continuous variables. We extracted the concordance and log-rank p-values for each model. We also compared the goodness of fit of each model compared to the null model with ANOVA. We extracted the chi-square and p-values as an output of this analysis.

### 2.6 Gene signature and gene correlation analysis

We analyzed the correlation of the 18 gene list with Oncotype Dx, EndoPredict, or MammaPrint signatures in TCGA breast cancer data using the correlation analysis tool of GEPIA2 (http://gepia2.cancer-pku.cn) (30). The Pearson method was used for correlation analysis. The housekeeping/reference genes in the signatures were excluded from the analysis. The correlation of the 18 gene list and Oncotype Dx, and EndoPredict signatures with *ESR1, ERBB2*, or *ESR1* plus *ERBB2* were also analyzed in GEPIA2. For the Oncotype Dx signature, the *ESR1, ERBB2*, or *ESR1* plus *ERBB2* was not included in the signature when correlation with *ESR1, ERBB2*, or *ESR1* plus *ERBB2* was analyzed respectively.

To investigate the correlation between the expression of each gene in the 18 gene list and Oncotype Dx genes, we extracted the data for 316 ER+/HER2- breast cancer patients who underwent neoadjuvant chemotherapy on the Kaplan-Meier Plotter database. We performed the hierarchical clustering analysis of the correlation matrices on Image GP using both the Pearson and the Spearman methods (http://www.ehbio.com/ImageGP) (31). Additionally, we extracted the data for correlation coefficients between all signature genes in all breast cancer patients (n=1100), patients with luminal A-type (n=568), and luminal B-type (n=219) breast cancer patients in the TCGA dataset from TIMER.2.0 (http://timer.cistrome.org) (32). We performed the hierarchical clustering analysis of the correlation matrices on Image GP using the Spearman method.

## 3 Results

### 3.1 Oncogenic signatures and upregulated genes associated with resistance to taxane-based neoadjuvant chemotherapy

To identify the markers associated with resistance to taxane-based neoadjuvant chemotherapy in ER+/HER2- breast cancer, we analyzed GSE20194, GSE20271, GSE25055, GSE25065, and GSE32646 datasets in GEO2R. These datasets included gene profiling data from breast cancers with different subtypes (19–25). We included and analyzed a total of 468 chemoresistant and 45 chemosensitive patients with ER+/HER2- breast cancer who underwent taxane-based neoadjuvant chemotherapy. We considered pathological complete response (pCR) as the surrogate of chemosensitivity and residual disease (RD) as the surrogate of chemoresistance. **Supplementary Table 1** lists the number of patients with RD or pCR in each dataset, with information on the source of samples, PAM50 intrinsic classes, and chemotherapy regimens.

**Figure 2:**
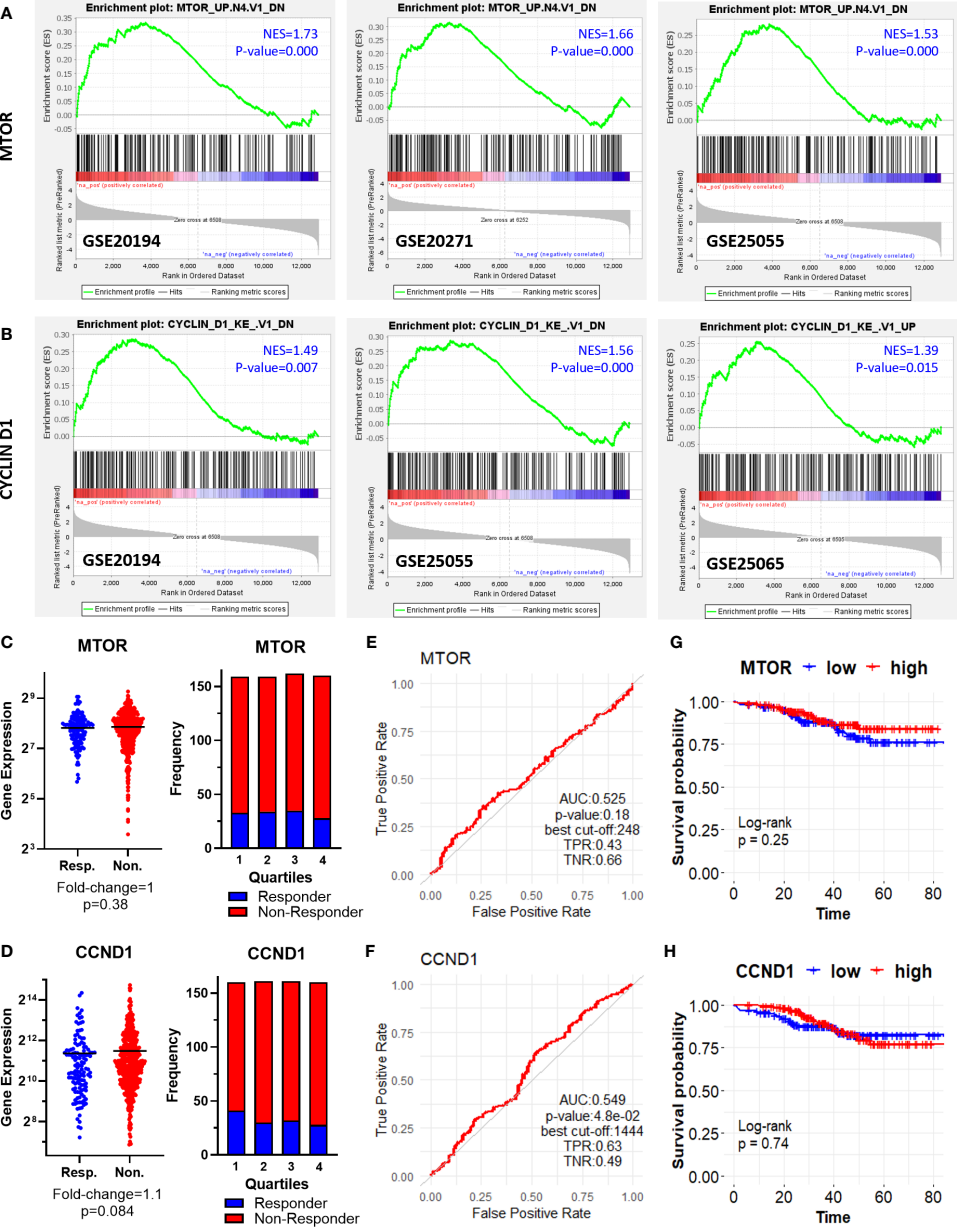
Oncogenic signature genes enriched in ER+/HER2- breast cancer patients resistant to taxane-based neoadjuvant chemotherapy. GSEA enrichment plots for **(A)** “MTOR_UP.N4.V1_DN” and **(B)** “CYCLIN_D1.KE.V1.DN” oncogenic signature gene sets in GSE20194, GSE20271, GSE25055, and GSE25065 datasets. The differential expression plot (left), and the frequency of responders and non-responders at each quartile of gene expression (right) for **(C)** MTOR and **(D)** CCND1; and the ROC plots for **(E)** MTOR and **(F)** CCND1 in 437 non-responsive vs. 75 responsive ER+/HER2- breast cancer patients who received taxane-based chemotherapy (ROC Plotter database). The pathological response was used as the surrogate of response to chemotherapy. The KM plots for **(G)** MTOR and **(H)** CCND1 in 316 ER+/HER2- breast cancer patients who received taxane-based neoadjuvant chemotherapy (KM Plotter database). AUC, Area under the curve; TPR, true positive rate; TNR, true negative rate; NES, normalized enrichment score.

First, we performed gene set enrichment analysis for each dataset to find out hallmark genes and oncogene signatures commonly enriched in patients resistant to therapy. We utilized 50 hallmark gene sets, and 189 oncogenic signature gene sets from Molecular Signatures Database (MSigDB) (27–29). Although we could not detect a hallmark gene set commonly enriched in all 5 GEO datasets, oncogenic signature gene sets “MTOR UP.N4.V1_DN” and “CYCLIN_D1.KE.V1.DN” were enriched at least in 3 out of 5 GEO datasets (**Figures 2A, B**).

“MTOR_UP.N4.V1_DN” signature consists of genes downregulated upon treatment of CEM-C1 T cell leukemia cells with an MTOR inhibitor rapamycin (33). “CYCLIN_D1.KE.V1.DN” gene signature includes genes downregulated in MCF-7 breast cancer cells heterogeneously over-expressing a mutant form of Cyclin D1 (K112E) lacking the ability to activate cyclin-dependent kinase 4 (CDK4) (34). Based on the significance of MTOR and CCND1 in tumor progression and resistance to therapy in cancer including breast cancer (35–38), we investigated whether *MTOR* and *CCND1* have a predictive significance for the pathological complete response to taxane-based chemotherapy, in 437 non-responsive vs. 75 responsive ER+/HER2- breast cancers patients in the ROC Plotter cohort. These two genes were not differentially expressed in non-responsive patients (**Figures 2C, D**), nor exhibit a predictive power in ROC analysis (**Figures 2E, F**). High expression of these genes in 316 ER+/HER2- breast cancer patients who had undergone neoadjuvant chemotherapy was not associated with a decreased relapse-free survival in the KM Plotter cohort (**Figures 2G, H**).

**Figure 3:**
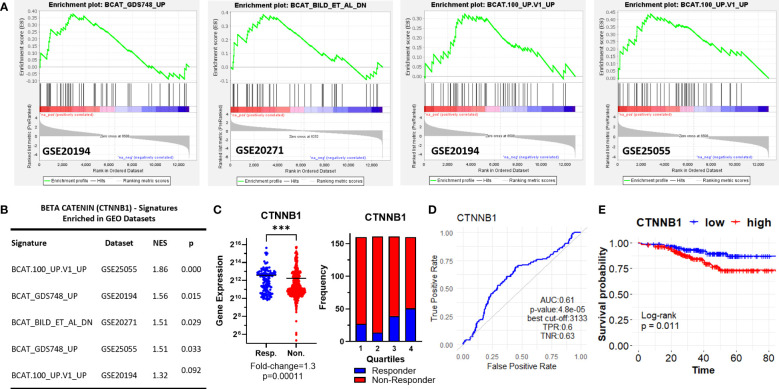
β-Catenin-related oncogenic signature genes enriched in ER+/HER2- breast cancer patients resistant to taxane-based therapy. **(A)** GSEA enrichment plots for β-Catenin-related oncogenic signature genes in GSE20194, GSE20271, and GSE25055 datasets with **(B)** the table of statistical parameters. **(C)** The differential expression (left), the frequency of responders and non-responders at each quartile of gene expression (right), and **(D)** the ROC curves for CTNNB1 in 437 non-responsive vs. 75 responsive ER+/HER2- breast cancer patients who received taxane-based chemotherapy (ROC Plotter database). **(E)** The KM plot for CTNNB1 in 316 ER+/HER2- breast cancer patients who received taxane-based neoadjuvant chemotherapy (KM Plotter database). AUC, Area under the curve; TPR, true positive rate; TNR, true negative rate; NES, normalized enrichment score.

Interestingly, we observed three different β-Catenin (CTNNB1)-related oncogenic signatures, “BCAT_GDS748_UP”, “BCAT.100_UP.V1_UP” “BCAT_BILD_ET_AL _DN” enriched in GSE20194, GSE20271, and GSE25055 datasets, although a single β-Catenin-related oncogenic signature is not commonly enriched among the datasets (**Figures 3A, B**). “BCAT_GDS748_UP” and “BCAT.100_UP.V1_UP” signatures consist of genes upregulated in HEK293 cells which express a constitutively active β-Catenin (39). “BCAT_BILD_ET_AL _DN” is established from the down-regulated genes in a primary epithelial breast cancer cell model that overexpresses active β-Catenin (40). β-Catenin is a crucial component of E-cadherin-mediated cell-cell adhesion and canonical WNT pathway which has high significance in mammary tissue development, breast cancer formation, and metastasis (41). Unexpectedly, our analysis of ER+/HER2- breast cancer patients in the ROC plotter cohort exhibited down-regulation of β-Catenin in patients with residual disease (**Figure 3C**). Despite that, the genes that are upregulated or downregulated in the presence of constitutively active CTNNB1 are enriched in non-responders in GEO datasets (**Figures 3A, B**), *CTNNB1* exhibited a significant predictive value in the ROC analysis (**Figure 3D**), and high expression of the *CTNNB1* was associated with decreased relapse-free survival in ER+/HER2- breast cancer patients who received neoadjuvant chemotherapy (**Figure 3E**). Since relapse-free survival is a better surrogate for response to therapy we evaluated these results in favor of the possible involvement of *CTNNB1* in resistance to taxane-based chemotherapy. These results also suggested the importance of the activity status of CTNNB1 besides the gene expression levels.

Then we explored upregulated genes and ontologies in chemoresistant patients in GEO datasets (**Supplementary Figures 1A-E**). A low number of upregulated genes were shared in all 5 datasets (1 gene: *XIST*), or 4 datasets (4 genes: *MLH3, TNFRSF25, SNX1, RBM5*). However, the overlap between the ontologies that the upregulated genes enriched was high in all 5 datasets (**Supplementary Figure 1F**). To investigate and compare the potential predictive power of a larger list of genes, we compiled the list of genes upregulated in 3 or more datasets, which included 63 coding genes.

### 3.2 Functionally enriched gene ontologies and pathways associated with resistance to taxane-based neoadjuvant chemotherapy

To identify the pathways and gene ontologies at which the 63 upregulated genes were enriched, we performed functional annotation and clustering analysis in The Database for Annotation, Visualization, and Integrated Discovery (DAVID) (Version 6.8) (https://david.ncifcrf.gov/) (42). The 63 upregulated genes clustered in 5 annotation clusters. The top annotation cluster with the highest enrichment score included “protein kinase activity” and “protein phosphorylation” as the most prominent biological processes and molecular functions (**Supplementary Table 2**). Ontologies related to “endocytosis” and “regulation of transcription” were the other prominent processes and molecular functions that the upregulated genes enriched in other clusters.

### 3.3 Validation of the differential expression and predictive power of upregulated genes

**Figure 4:**
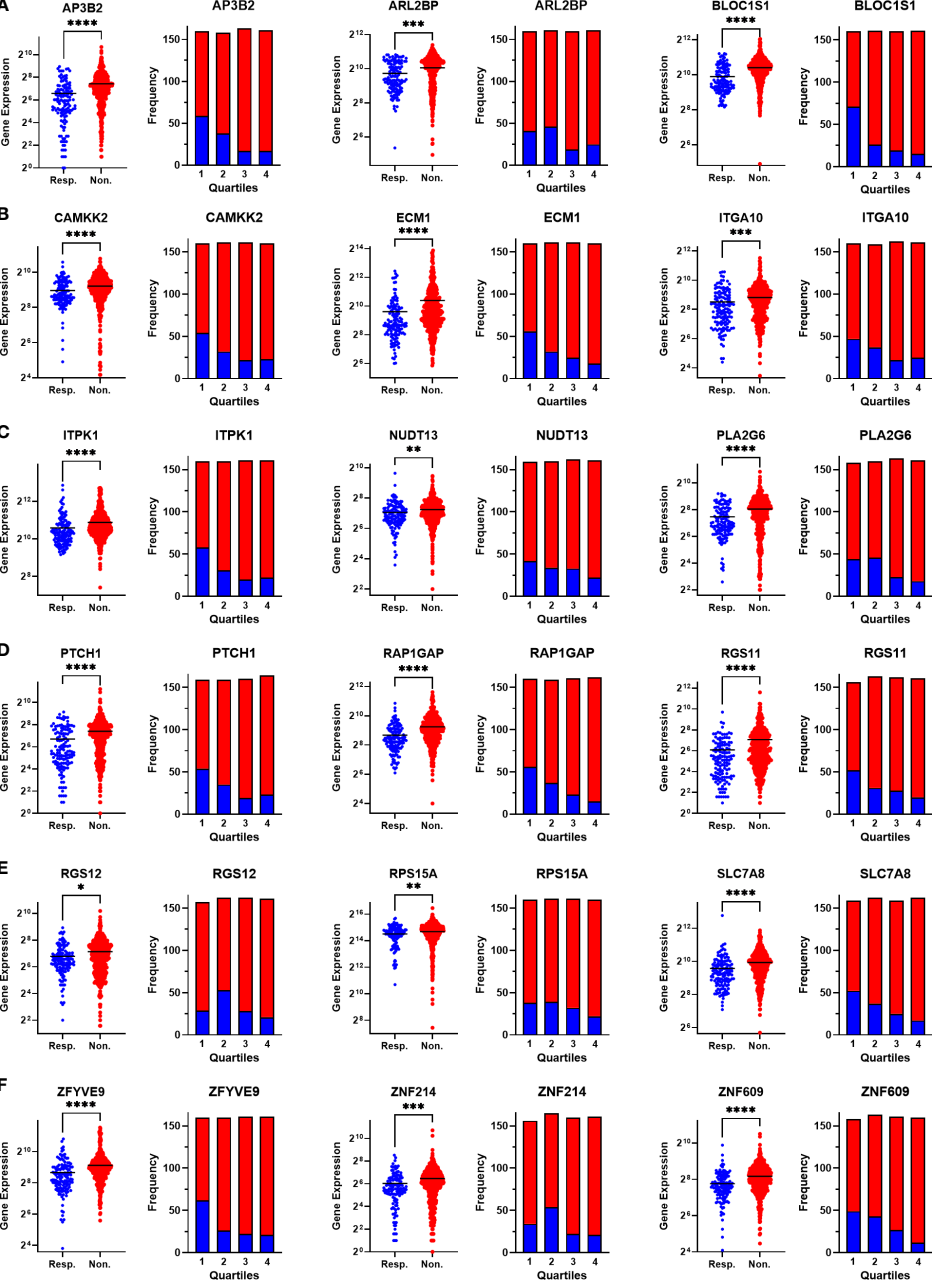
Validating the differential expression of the 18 upregulated genes in non-responders. The differential expression (left), and the frequency of responders and non-responders at each quartile of gene expression (right) for **(A)** AP3B2, ARL2BP, BLOC1S1, **(B)** CAMKK2, ECM1, and ITGA10, **(C)** ITPK1, NUDT13, PLA2G6, **(D)** PTCH1, RAP1GAP, and RGS11, **(E)** RGS12, RPS15A, SLC7A8, **(F)** ZFYVE9, ZNF214, and ZNF609. The analysis was performed on data for 437 non-responsive vs. 75 responsive ER+/HER2- breast cancer patients who received taxane-based chemotherapy in the ROCplotter cohort. The pathological response was used as the surrogate of response to chemotherapy. Resp.: Responders (blue), Non.:Non-responders (red). *: p<0.05, **: p<0.01, ***: p<0.001, ****: p<0.0001.

**Figure 5:**
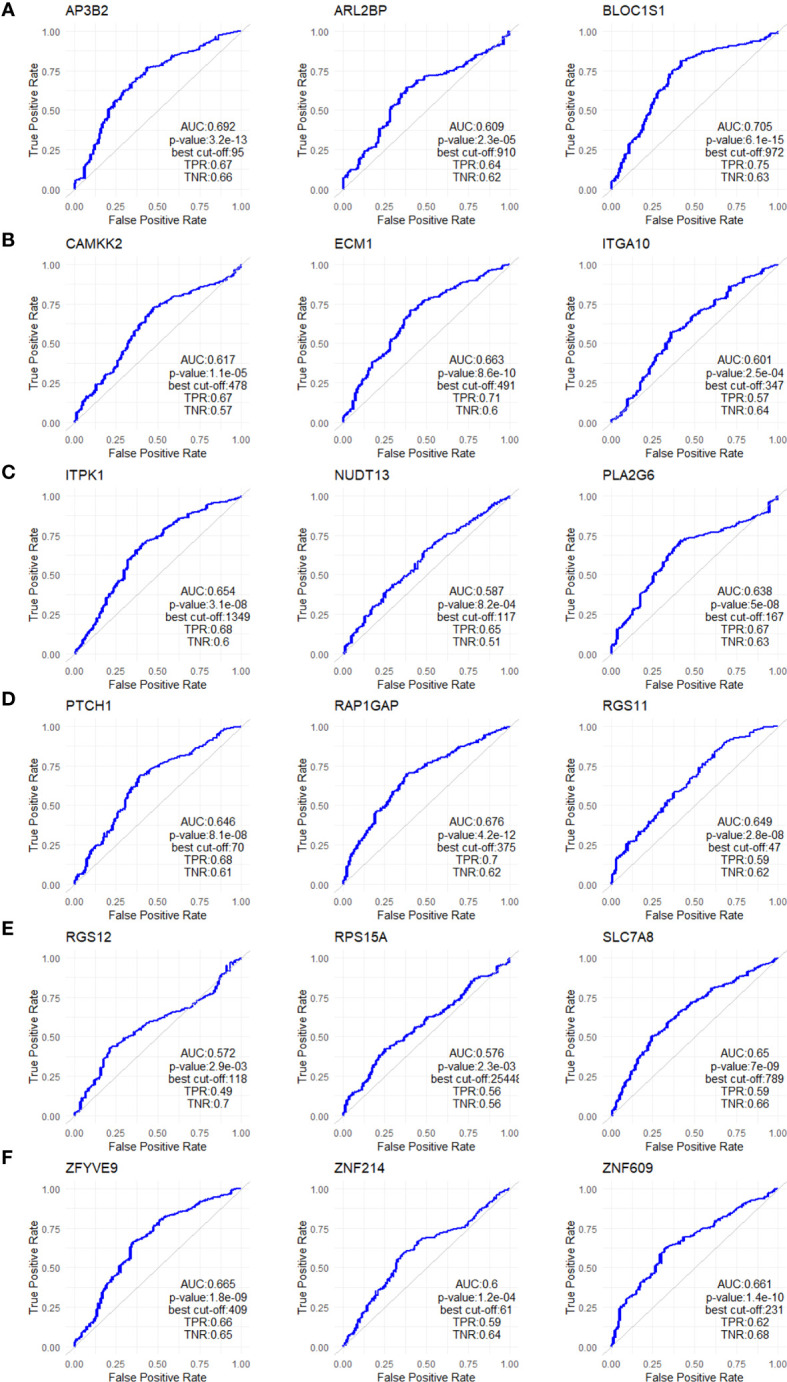
ROC analysis of the 18 upregulated genes in non-responders. ROC plots for **(A)** AP3B2, ARL2BP, BLOC1S1, **(B)** CAMKK2, ECM1, and ITGA10, **(C)** ITPK1, NUDT13, PLA2G6, **(D)** PTCH1, RAP1GAP, and RGS11, **(E)** RGS12, RPS15A, SLC7A8, **(F)** ZFYVE9, ZNF214, and ZNF609. The analysis was performed on data for 437 non-responsive vs. 75 responsive ER+/HER2- breast cancer patients who received taxane-based chemotherapy in the ROCplotter cohort. The pathological response was used as the surrogate of response to chemotherapy.

To validate the upregulation of the 63 genes in chemoresistant patients and investigate their predictive value for resistance to taxane-based chemotherapy, we analyzed their differential expression and ROC plots in a large cohort of breast cancer patients in the ROC plotter developed by Fekete and Győrffy (16). Among these 63 genes, we validated the significant upregulation of 18 genes in non-responders to taxane-based chemotherapy (**Figure 4**). The ROC plots of these 18 genes also demonstrated a statistically significant power to predict resistance to taxane-based therapy in 437 non-responsive vs. 75 responsive ER+/HER2- breast cancer patients (**Figure 5**). The area under the ROC curves (AUC) was significantly higher than 0.5 for all 18 genes (p<0.01 for 2 genes and p<0.001 for 16 genes). These genes with functional annotations are listed in **Supplementary Table 3**. Among the 18 validated genes, *BLOC1S1, AP3B2, ZNF609, ZFYVE9*, and *RAP1GAP* were the top 5 genes with the highest PPV and NPV (**Table 1**).

**Table 1:** The predictive power of the 18 upregulated genes in different breast cancer subtypes.

	ER +/HER2 -				HER2 +/ER -				TNBC			
Genes	AUC	P-value	PPV	NPV	AUC	P-value	PPV	NPV	AUC	P-value	PPV	NPV
**AP3B2**	**0.692**	**3.2E-13**	**0.663**	**0.667**	0.623	4.3E-03	0.625	0.598	0.596	2.3E-03	0.580	0.580
**ARL2BP**	**0.609**	**2.3E-05**	**0.627**	**0.633**	0.537	2.2E-01	0.600	0.574	0.523	2.5E-01	0.564	0.541
**BLOC1S1**	**0.705**	**6.1E-15**	**0.670**	**0.716**	0.604	1.5E-02	0.624	0.626	0.587	5.3E-03	0.584	0.586
**CAMKK2**	**0.617**	**1.1E-05**	**0.609**	**0.633**	0.545	1.8E-01	0.632	0.568	0.539	1.3E-01	0.564	0.557
**ECM1**	**0.663**	**8.6E-10**	**0.640**	**0.674**	0.502	4.8E-01	0.568	0.540	0.550	7.3E-02	0.608	0.570
**ITGA10**	**0.601**	**2.5E-04**	**0.613**	**0.598**	0.591	2.9E-02	0.632	0.581	0.557	5.0E-02	0.563	0.549
**ITPK1**	**0.654**	**3.1E-08**	**0.630**	**0.652**	0.614	7.7E-03	0.588	0.616	0.552	6.2E-02	0.573	0.559
**NUDT13**	**0.587**	**8.2E-04**	**0.570**	**0.593**	0.623	4.4E-03	0.639	0.663	0.585	5.3E-03	0.567	0.563
**PLA2G6**	**0.638**	**5.0E-08**	**0.644**	**0.656**	0.553	1.4E-01	0.598	0.568	0.523	2.5E-01	0.528	0.532
**PTCH1**	**0.646**	**8.1E-08**	**0.636**	**0.656**	**0.666**	**1.6E-04**	**0.663**	**0.667**	0.597	2.1E-03	0.576	0.627
**RAP1GAP**	**0.676**	**4.20E-12**	**0.648**	**0.674**	0.569	7.6E-02	0.578	0.607	0.532	1.8E-01	0.542	0.561
**RGS11**	**0.649**	**2.8E-08**	**0.608**	**0.602**	0.577	5.5E-02	0.588	0.630	**0.627**	**6.4E-05**	**0.586**	**0.584**
**RGS12**	0.572	2.9E-03	0.620	0.579	0.503	4.7E-01	0.587	0.574	0.564	3.0E-02	0.571	0.569
**RPS15A**	0.576	2.3E-03	0.560	0.560	0.532	2.6E-01	0.570	0.545	0.561	3.6E-02	0.563	0.580
**SLC7A8**	**0.65**	**7.0E-09**	**0.634**	**0.617**	0.560	1.0E-01	0.600	0.560	0.561	3.6E-02	0.549	0.551
**ZFYVE9**	**0.665**	**1.8E-09**	**0.653**	**0.657**	0.556	1.2E-01	0.579	0.620	**0.611**	**4.9E-04**	**0.607**	**0.636**
**ZNF214**	**0.6**	**1.2E-04**	**0.621**	**0.610**	0.531	2.7E-01	0.519	0.521	0.551	6.8E-02	0.552	0.548
**ZNF609**	**0.661**	**1.4E-10**	**0.660**	**0.642**	0.621	4.9E-03	0.600	0.611	0.595	2.2E-03	0.577	0.573

The results for which the p-value for AUC was 0.001<p<0.05 were underlined, and p<0.001 were written in bold. Number of patients: ER+/HER2-: non-responders: 437, responders: 75; HER2+/ER-: non-responders: 31, responders:40; TNBC: non-responders:125, responders:79. Analysis was performed on ROC Plotter database.

### 3.4 The specificity of the 18 upregulated genes to ER+/HER2- breast cancer

Breast cancer is a heterogeneous disease with different responses to treatment in different subtypes (4, 7). Therefore, distinct gene lists may have different predictive strengths in different subtypes. To test whether the predictive value of the 18 upregulated genes we identified is specific to the ER+/HER2- breast cancer, we tested the predictive power of each gene also in HER2+/ER- and triple-negative breast cancers (**Table 1**).

Among the 18 genes, fewer genes exhibited significant predictive value in HER2+ cancers and TNBC, compared with ER+/HER2- breast cancer. Despite the p-values for AUCs of some genes such as *AP3B2, BLOC1S1, NUDT13, PTCH1*, and *ZNF609* being statistically significant in all three breast cancer subtypes, their significance in HER2+ cancers and TNBC were much lower compared to that in ER+/HER2- breast cancer patients. PPV and NPVs were also lower compared to that in ER+/HER2- breast cancer. These results showed that the 18 gene signature has higher predictive value and specificity in ER+/HER2- breast cancer.

### 3.5 Comparative analysis of the 18 upregulated genes with prognostic signatures in breast cancer

**Figure 6:**
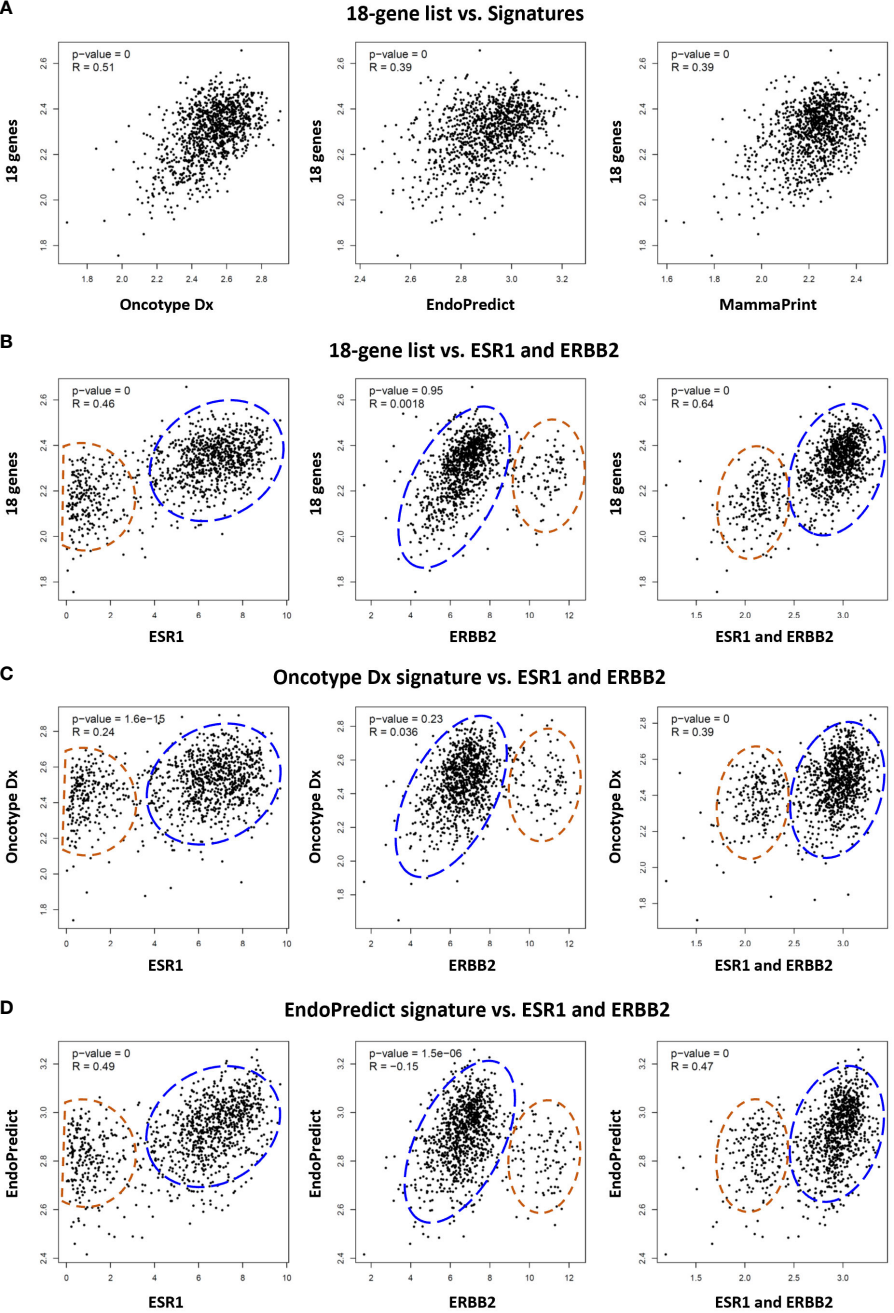
Comparative analysis of the 18 gene list with multi-gene assay signatures in TCGA breast cancer patients. **(A)** Correlation between the 18 gene list and Oncotype Dx, EndoPredict, and MammaPrint signatures respectively in 1100 breast cancer patients in TCGA dataset. Correlation of **(B)** 18 gene list, **(C)** Oncotype Dx, and **(D)** EndoPredict signatures with ER (ESR1), HER2 (ERBB2), and ER plus ERBB2 respectively in breast cancer patients. The housekeeping/reference genes in all the signatures were excluded from the analysis. For the Oncotype Dx signature, the ESR1, ERBB2, or ESR1 plus ERBB2 was not included in the signature when correlation with ESR1, ERBB2, or ESR1 plus ERBB2 was analyzed respectively in (6c). The blue dashed circles show the main cluster of patients with high expression of 18-genes or two signatures in the y-axis, together with high ESR1 expression (left graph), low ERBB2 expression (middle), and high ESR1+ERBB2 expression (right graph) in the x-axis. The red dashed circles show patients with lower expression of the 18 genes or two signatures in the y-axis, and low ESR1(left), high ERBB2 (middle), and low ESR1+ERBB2 (right) expression in the x-axis. x- and y-axis represent log(transcript per million) values for gene expression.

Several multi-gene assays such as Oncotype Dx, EndoPredict, and MammaPrint are being used as complementary tools to guide decisions in the clinical management of breast cancer patients. Although their primary benefit is to estimate the risk of recurrence after endocrine therapy in ER+/HER2- breast cancers, some studies suggested their utility also as predictive tools to estimate response to systemic chemotherapy (10, 43–45). Therefore, we investigated the correlation of our 18 gene list with these signatures. The analysis of TCGA dataset demonstrated that the expression of the 18 genes is correlated with the expression of Oncotype Dx, EndoPredict, and MammaPrint signatures in 1100 breast cancer patients. The correlation of the 18 gene list was highest with the Oncotype Dx (**Figure 6A**).

Then we compared the correlation of the 18 genes, Oncotype Dx and EndoPredict signatures with the expression of ER (*ESR1*), HER2 (*ERBB2*), and ER plus HER2 in breast cancer patients in TCGA dataset. The 18 genes and the two signatures were correlated with the expression of ER and ER plus HER2 in breast cancer patients (**Figures 6B–D**). The correlation with HER2 was significant only for the EndoPredict signature. It was noticeable that the clustering pattern of patients in correlation analysis with ER, HER2, and ER plus HER2 were very similar for the 18 gene list (**Figure 6B**) and the two signatures (**Figures 6C, D**). Since the correlation of the 18 genes was highest with Oncotype Dx (**Figure 6A**), and the Oncotype Dx signature bears a comparable number of genes (16 marker genes + 5 reference genes), we further analyzed the characteristics of our 18 gene list comparatively with Oncotype Dx.

### 3.6 Comparative analysis of the 18 gene list with the Oncotype Dx signature in breast cancer

Despite that multi-gene signatures are used to calculate recurrence scores based on multivariate statistical equations (8), we wondered whether the individual predictive powers of the 18 gene list we identified are similar to the individual predictive powers of Oncotype Dx genes in different subtypes of breast cancer. Hence, we investigated the individual predictive powers of Oncotype Dx genes in three breast cancer subtypes in the ROC plotter database. The AUCs were significantly higher than 0.5 for only 8 genes (p<0.05 for 2 genes and p<0.001 for 6 genes) in ER+/HER2- breast cancer patients who received taxane-based chemotherapy (**Table 2**). *BCL2*, *ERBB2*, *GRB7*, and *SCUBE2* exhibited highest predictive strength. *SCUBE2* also emerged as a key predictor of chemoresistance in breast cancer in our recent study (46). Only one or two Oncotype Dx genes exhibited predictive value in HER2+/ER- and TNBC subtypes, suggesting specificity of the signature for the ER+/HER2- subtype. However, nearly half of the Oncotype Dx genes did not exhibit a predictive value in ER+/HER2- breast cancer.

**Table 2:** The predictive power of Oncotype Dx genes in different breast cancer subtypes.

	ER +/HER2 -				HER2 +/ER -				TNBC			
Genes	AUC	P-value	PPV	NPV	AUC	P-value	PPV	NPV	AUC	P-value	PPV	NPV
**AURKA**	0.579	3.5E-03	0.588	0.616	0.527	2.9E-01	0.548	0.542	0.503	4.6E-01	0.523	0.527
**BAG1**	0.558	1.6E-01	0.677	0.585	0.646	1.1E-02	0.757	0.638	0.531	3.3E-01	0.596	0.585
**BCL2**	**0.701**	**3.7E-14**	**0.651**	**0.670**	0.559	1.1E-01	0.587	0.594	0.553	6.1E-02	0.545	0.545
**BIRC5**	0.513	3.3E-01	0.535	0.526	0.502	4.8E-01	0.546	0.554	0.51	3.8E-01	0.538	0.532
**CCNB1**	0.574	7.7E-02	0.596	0.628	0.503	4.8E-01	0.576	0.537	0.596	9.4E-02	0.621	0.593
**CD68**	0.531	1.4E-01	0.535	0.535	0.561	1.0E-01	0.571	0.622	0.537	1.4E-01	0.584	0.553
**CTSL2**	**0.6**	**1.5E-04**	**0.605**	**0.571**	0.545	1.8E-01	0.619	0.555	0.544	1.0E-01	0.552	0.558
**ERBB2**	**0.644**	**2.3E-08**	**0.626**	**0.645**	0.551	4.1E-01	0.569	0.533	0.511	3.7E-01	0.521	0.519
**ESR1**	**0.595**	**6.2E-04**	**0.571**	**0.569**	0.555	1.3E-01	0.582	0.554	0.582	8.1E-03	0.624	0.591
**GRB7**	**0.646**	**1.7E-08**	**0.657**	**0.663**	0.54	2.1E-01	0.566	0.574	0.571	1.8E-02	0.588	0.565
**GSTM1**	NA	NA	NA	NA	NA	NA	NA	NA	NA	NA	NA	NA
**MKI67**	0.506	4.2E-01	0.513	0.517	0.522	3.3E-01	0.571	0.552	0.506	4.3E-01	0.546	0.544
**MMP11**	0.531	1.5E-01	0.527	0.523	0.562	9.9E-02	0.545	0.545	0.532	1.8E-01	0.548	0.579
**MYBL2**	0.549	3.9E-02	0.545	0.556	0.52	3.4E-01	0.524	0.517	0.528	2.0E-01	0.538	0.533
**PGR**	0.531	2.9E-01	0.568	0.554	0.53	3.4E-01	0.571	0.579	0.599	8.5E-02	0.638	0.653
**SCUBE2**	**0.628**	**6.7E-07**	**0.590**	**0.641**	0.557	1.2E-01	0.581	0.561	0.534	1.6E-01	0.538	0.532

The results for which the p-value for AUC was 0.001<p<0.05 were underlined, and p<0.001 were written in bold. The housekeeping/reference genes ACTB, GAPDH, GUSB, RPLP0, and TFRC in Oncotype Dx signature were excluded from the analysis, and the data was not available (NA) for GSTM1. Number of patients: ER+/HER2-:non-responders: 437, responders: 75; HER2+/ER-: non-responders: 31, responders:40; TNBC: non-responders:125, responders:79.

Then we compared the differential expression of the 18 gene list and Oncotype Dx signature in chemoresistant patients with different breast cancer subtypes in the ROC plotter dataset. All the genes in the 18 gene list were significantly increased in the ER+/HER2- subtype and most of the fold changes (FCs) in HER2+/ER- and TNBC subtypes were insignificant (**Table 3**). The FC for almost all the Oncotype Dx genes was also insignificant in HER2+/ER- and TNBC subtypes. However, only 7 out of 16 Oncotype Dx genes were significantly upregulated in ER+/HER2- breast cancer. These results, together with the comparison of the results in **Tables 1**, **2** suggest that the 18 genes we identified exhibit higher individual predictive values and higher specificity to ER+/HER2- breast cancer patients.

**Table 3:** The differential expression of 18 gene list vs. Oncotype Dx signature genes in taxane-resistant patients with different breast cancer subtypes.

18-gene	ER +/HER2 -		HER2 +/ER -		TNBC		Oncotype Dx	ER +/HER2 -		HER2 +/ER -		TNBC	
Genes	FC	P-value	FC	P-value	FC	P-value	Genes	FC	P-value	FC	P-value	FC	P-value
AP3B2	1.8	1.2E-11	1.3	1.1E-02	1.3	5.2E-03	AURKA	1.2	5.2E-03	1.1	5.8E-01	1	9.2E-01
ARL2BP	1.5	1.2E-06	1.1	4.4E-01	1.1	5.1E-01	BAG1	1.2	2.9E-01	1.7	3.4E-02	1.3	6.8E-01
BLOC1S1	1.4	3.9E-13	1.1	3.3E-02	1.1	1.1E-02	BCL2	2.1	1.2E-12	1	2.3E-01	1.2	1.3E-01
CAMKK2	1.2	4.0E-04	1.1	3.5E-01	1.1	2.6E-01	BIRC5	1	6.4E-01	1	9.6E-01	1	7.7E-01
ECM1	1.2	5.3E-08	1.2	9.7E-01	1.1	1.5E-01	CCNB1	1.3	1.8E-01	1	9.7E-01	1.5	2.1E-01
ITGA10	1.2	3.8E-04	1.3	6.0E-02	1.1	9.4E-02	CD68	1.1	2.8E-01	1.2	2.1E-01	1.3	2.8E-01
ITPK1	1.6	2.6E-07	1.2	1.9E-02	1.1	1.3E-01	CTSL2	1.2	4.3E-04	1.1	3.6E-01	1.1	2.0E-01
NUDT13	1.1	7.4E-03	1.3	1.1E-02	1.3	1.3E-02	ERBB2	1.3	3.3E-07	1	8.2E-01	1.1	7.4E-01
PLA2G6	1.3	1.4E-08	1.3	2.7E-01	1.1	5.0E-01	ESR1	1.2	8.3E-04	1.1	2.5E-01	1.1	1.7E-02
PTCH1	1.3	1.2E-07	1.6	6.4E-04	1.4	4.6E-03	GRB7	1.5	2.6E-07	1	4.1E-01	1.2	4.0E-02
RAP1GAP	1.7	7.6E-09	1.2	1.5E-01	1.0	3.5E-01	GSTM1	NA	NA	NA	NA	NA	NA
RGS11	1.1	2.1E-03	1.1	1.1E-01	1.7	2.1E-04	MKI67	1.1	8.2E-01	1	6.5E-01	1	8.7E-01
RGS12	1.3	1.2E-04	1.0	9.5E-01	1.2	6.2E-02	MMP11	1	2.8E-01	1.5	2.0E-01	1.1	3.5E-01
RPS15A	1.3	1.1E-02	1.1	5.1E-01	1.1	7.6E-02	MYBL2	1.1	8.2E-02	1	6.9E-01	1.1	4.1E-01
SLC7A8	2	1.4E-07	1.2	2.1E-01	1.2	7.5E-02	PGR	1	5.8E-01	1	6.6E-01	1.1	1.9E-01
ZFYVE9	1.4	5.0E-09	1.3	2.5E-01	1.3	1.2E-03	SCUBE2	1.7	6.0E-06	1.1	2.4E-01	1.3	3.3E-01
ZNF214	1.2	3.6E-05	1.3	5.3E-01	1.4	1.4E-01							
ZNF609	1.5	4.8E-10	1.2	1.3E-02	1.3	5.5E-03							

The results for which the p-value for FC (fold-change) was 0.001<p<0.05 were underlined, and p<0.001 were written in bold. The housekeeping/reference genes ACTB, GAPDH, GUSB, RPLP0, and TFRC in Oncotype Dx signature were excluded from the analysis, and the data was not available (NA) for GSTM1. Number of patients: ER+/HER2-:non-responders: 437, responders: 75; HER2+/ER-: non-responders: 31, responders:40; TNBC: non-responders:125, responders:79.

**Figure 7:**
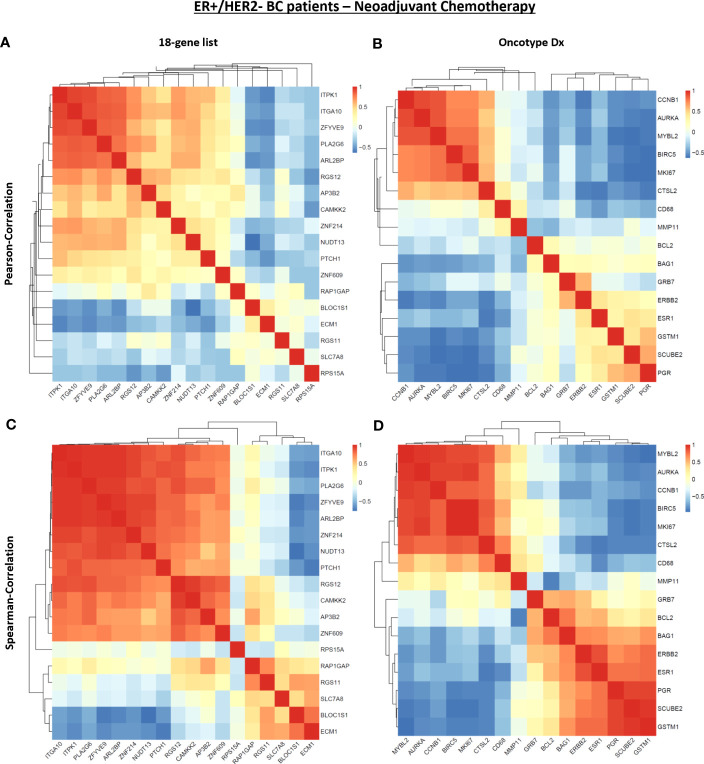
The correlation between genes in the 18 gene list and the Oncotype Dx signature in ER+/HER2- breast cancer patients. Pearson correlation between the expression of **(A)** 18 genes and **(B)** Oncotype Dx genes in 316 ER+/HER2- breast cancer patients who underwent neoadjuvant chemotherapy (KM plotter database). Spearman correlation between the expression of **(C)** 18 genes and **(D)** Oncotype Dx genes in 316 ER+/HER2- breast cancer patients who underwent neoadjuvant chemotherapy (KM plotter database). The housekeeping/reference genes ACTB, GAPDH, GUSB, RPLP0, and TFRC in the Oncotype Dx signature were excluded from the analysis.

After that, we analyzed the Pearson correlation between each gene in the 18 gene list (**Figure 7A**) and the Oncotype Dx signature (**Figure 7B**) in 316 ER+/HER2- breast cancer patients who underwent neoadjuvant chemotherapy. The 18 gene list formed 3 main clusters: a cluster of positively correlated genes (*ITPK1*, *ITGA10*, *ZFYVE9*, *PLA2G6, ARL2BP*, and *RGS12*), a cluster of uncorrelated or poorly correlated genes (*AP3B2*, *CAMKK2, ZNF214, NUDT13, PTCH1*, and *ZNF609*) and a cluster of genes negatively correlated with others in the 18 gene-list (*RAP1GAP, BLOC1S1, ECM1, RGS11, SLCA78*, and *RPS15A*). Oncotype Dx genes also formed 3 main clusters but with a different pattern: a cluster of positively correlated genes (*CCNB1, AURKA, MYBL2, BIRC5, MKI67*, and *CTSL2*), a cluster of uncorrelated or poorly correlated genes (*CD68, MMP11, BCL2, BAG1*, and *GRB7*), and a second cluster of positively correlated genes (*ERBB2, ESR1, GSTM1, SCUBE2*, and *PGR*).

**Figure 8:**
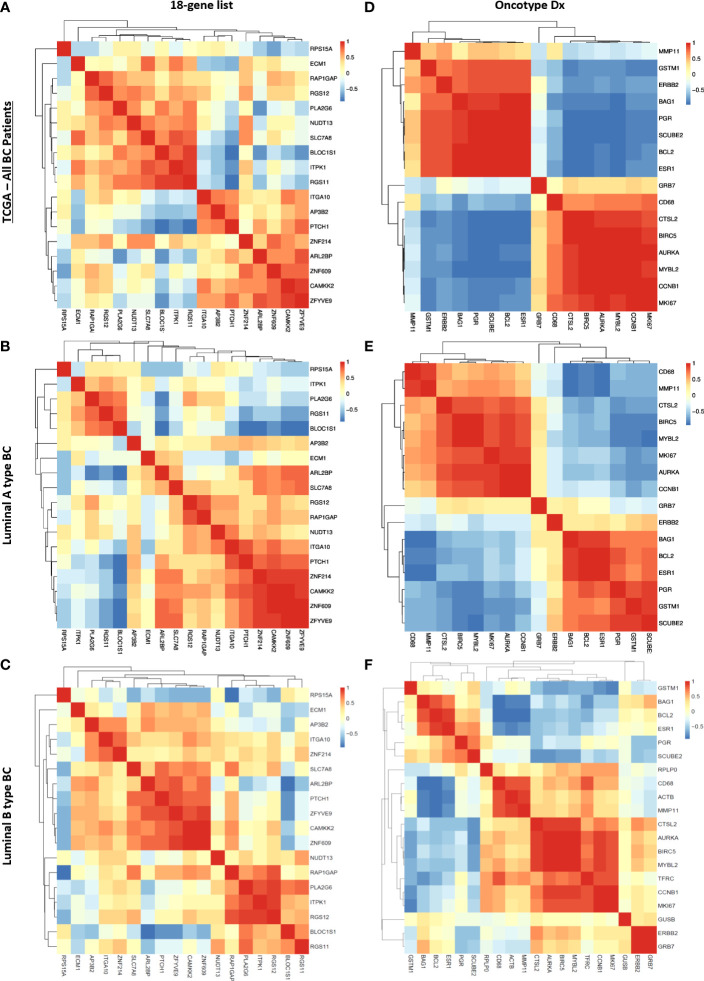
Correlation between genes in the 18 gene list and the Oncotype Dx signature in TCGA breast cancer patients. Spearman correlation heatmap of the expression of 18 genes in TCGA **(A)** all breast cancer patients (n=1100), **(B)** luminal-A type breast cancer patients (n=568), and **(C)** luminal-B type breast cancer patients (n=219). Spearman correlation heatmap of the expression of Oncotype Dx genes in TCGA **(D)** all breast cancer patients (n=1100), **(E)** luminal-A type breast cancer patients (n=568), and **(F)** luminal-B type breast cancer patients (n=219). The housekeeping/reference genes ACTB, GAPDH, GUSB, RPLP0, and TFRC in the Oncotype Dx signature were excluded from the analysis.

Although linear correlation for some genes is poor, they may exhibit concordant increases or decreases in expression in tumor samples. To investigate these kinds of monotonic relationships we analyzed the Spearman correlation between each gene in the 18 gene list (**Figure 7C**) and the Oncotype Dx signature (**Figure 7D**) in 316 ER+/HER2- breast cancer patients. The 18 genes constituted two main clusters of positively and monotonically correlated genes. The larger cluster included *ITGA10, ITPK1, PLA2G6, ZFYVE9, ARLB2BP, ZNF214, NUDT13, PTCH1, RGS12, CAMKK2, AP3B2*, and *ZNF609*. The smaller cluster consisted of *RAP1GAP, RGS11, SLC7A8, BLOC1S1*, and *ECM1* (**Figure 7C**). The Oncotype Dx signature exhibited 2 clusters of positively and monotonically changing clusters of similar size. These 2 clusters, one composed of *ESR1*-related genes, and the other composed of genes associated with proliferation like *MKI67* and *CCNB1* exhibited changes in the opposite direction in ER+/HER2- breast cancer patients (**Figure 7D**).To understand whether this pattern of correlation within the 18-gene list and Oncotype Dx is specific to ER+/HER2- breast cancer patients, we performed a similar Spearman correlation analysis in TCGA breast cancer data (**Figure 8**).

We observed that the pattern of correlation of 18 genes was different in all breast cancer patients in the TCGA dataset with lower correlation coefficients in general (**Figure 8A**) compared to that in the cohort of ER+/HER2- breast cancer patients (**Figure 7C**). The pattern of the Spearman correlation matrix was also different in luminal A- or luminal B- type breast cancers in the TCGA dataset (**Figures 8 B, C**). On the other hand, the Oncotype Dx signature exhibited nearly the same correlation pattern in all TCGA breast cancer patients and luminal A-type breast cancer patients (**Figures 8D, E**) as in the cohort of ER+/HER2- breast cancer patients (**Figure 7D**). The pattern in luminal B-type breast cancer was different from that in other cohorts (**Figure 8F**). These results suggested that the 18 gene list we identified may be more representative of ER+/HER2- breast cancer patients, while Oncotype Dx similarly represents all breast cancer subtypes, Luminal A type, and ER+/HER2- breast cancer.

### 3.7 The effect of 18 genes in the relapse free survival of ER+/HER2- breast cancer patients

**Figure 9:**
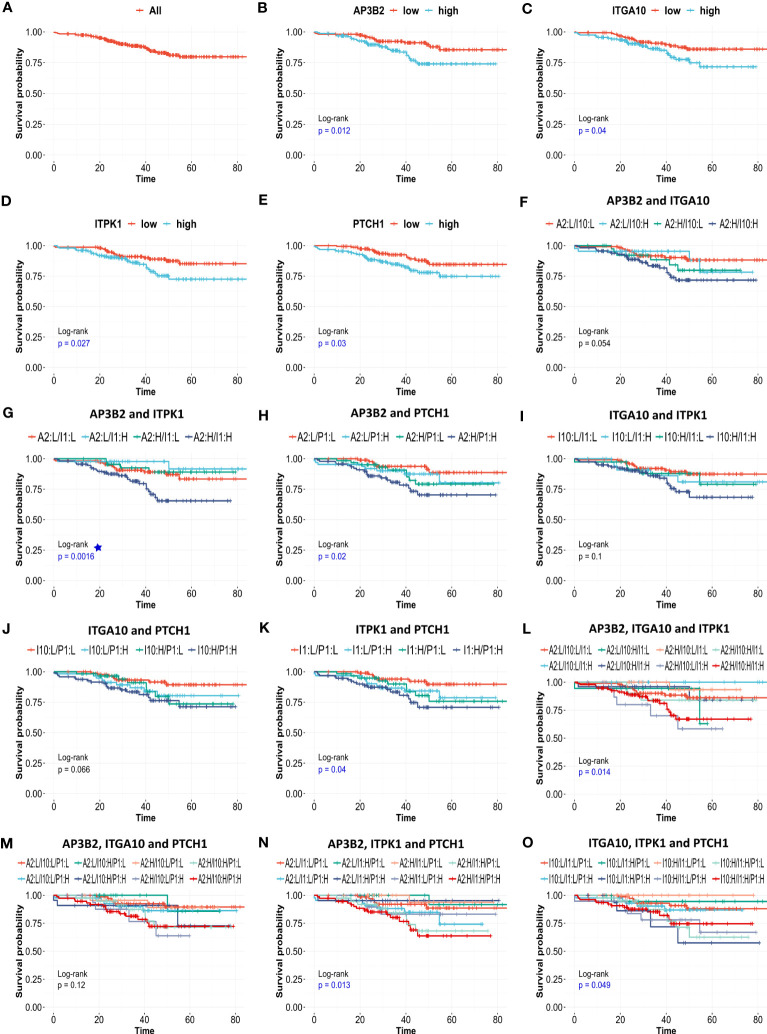
The effects of AP3B2, ITGA10, ITPK1, and PTCH1 on relapse-free survival of ER+/HER2- breast cancer patients. KM survival plots for the **(A)** null model, **(B)** AP3B2, **(C)** ITGA10, **(D)** ITPK1, **(E)** PTCH1, **(F)** AP3B2 and ITGA10, **(G)** AP3B2 and ITPK1, **(H)** AP3B2 and PTCH1, **(I)** ITGA10 and ITPK1, **(J)** ITGA10 and PTCH1, **(K)** ITPK1 and PTCH1, **(L)** AP3B2, ITGA10, and ITPK1, **(M)** AP3B2, ITGA10, and PTCH1, **(N)** AP3B2, ITPK1, and PTCH1, **(O)** ITGA10, ITPK1, and PTCH1, in 316 ER+/HER2- breast cancer patients who underwent neoadjuvant chemotherapy. H: high, L: low.

Pathological complete response is often used as a surrogate of treatment response to neoadjuvant chemotherapy. Since its assessment within the period of clinical studies is relatively easy, pCR gained widespread use as a primary endpoint in many clinical trials to facilitate and accelerate the drug discovery process. However, recent studies question its efficacy as a predictor of patient survival and reveal varying surrogacy of pCR in distinct breast cancer subtypes (47–49). To identify reliable predictors of resistance to neoadjuvant chemotherapy we investigated the effect of 18 genes in relapse-free survival of 316 ER-/HER2- patients in the KM plotter cohort. We validated that high expression of 4 out of 18 genes, namely *AP3B2, ITGA10, ITPK1*, and *PTCH1* is associated with a significantly decreased relapse-free survival (**Figures 9A–E**).

Then we performed multivariate survival analysis using these four genes as categorical variates (**Figures 9F–O**). The survival models were statistically significant for gene combinations *AP3B2/ITPK1*, *AP3B2/PTCH1, ITPK1/PTCH1, AP3B2/ITGA10/ITPK1, AP3B2/ITPK1/PTCH1*, and *ITGA10/ITPK1/PTCH1* (**Figures 9G, H, K, L, N, O**). *AP3B2/ITPK1* model remarkably had the greatest significance in the log-rank test (**Figure 9G**).

### 3.8 The effect of AP3B2, ITGA10, ITPK1, PTCH1 and CTNNB1 as continuous covariates in the relapse free survival of ER+/HER2- breast cancer patients

Despite that KM survival curves are efficient tools to assess the impact of gene expression on patient outcome, the cut-offs used to allocate patients to low-expression and high-expression groups substantially affect the results. To assess the impact of gene expression on patient outcomes independent of cut-offs, we performed Cox Proportional Hazards regression using *AP3B2, ITGA10, ITPK1*, and *PTCH1* as continuous covariates. We also included *CTNNB1* in the analysis since *CTNNB1*-related signatures were enriched in ER+/HER2- breast cancer patients resistant to taxane-based therapy and high expression of *CTNNB1* was associated with decreased survival in ER+/HER2- breast cancer patients (**Figures 3A, B, D**). We established univariate and multivariate Cox Proportional Hazards regression models of these genes using the relapse-free survival data of 316 ER+/HER2- Breast Cancer patients who underwent neoadjuvant chemotherapy (**Table 4**). Nearly half of the univariate and multivariate models fitted survival data significantly, and nearly all of them achieved significantly better fits compared to the null model in ANOVA.

**Table 4:** The statistical significance measures for Cox Proportional Hazards Regression Models.

Gene/Genes	COX-PH Model Fit			ANOVA		
	Concordance	Log-rank p	Sig.	Chi-Sqr	Pr(>|Chi|)	Sig.
AP3B2	0.559	1		0.0008	0.978	
ITGA10	0.556	0.4		0.8412	<2e-16	***
ITPK1	0.603	0.2		0.5873	<2e-16	***
PTCH1	0.625	0.01	*	6.4123	<2e-16	***
CTNNB1	0.602	0.02	*	2.4801	<2e-16	***
AP3B2 + ITGA10	0.540	0.5		4.0103	0.045	*
AP3B2 + ITPK1	0.597	0.4		0.4428	<2e-16	***
AP3B2 + PTCH1	0.630	0.02	*	4.975	<2e-16	***
ITGA10 + ITPK1	0.606	0.5		5.3101	<2e-16	***
ITGA10 + PTCH1	0.626	0.03	*	6.4228	<2e-16	***
ITPK1 + PTCH1	0.633	0.03	*	0.2529	<2e-16	***
AP3B2 + CTNNB1	0.624	0.05		2.3355	<2e-16	***
ITGA10 + CTNNB1	0.616	0.02	*	1.9464	<2e-16	***
ITPK1 + CTNNB1	0.625	0.04	*	0.6459	<2e-16	***
PTCH1 + CTNNB1	0.660	0.003	**	6.7732	<2e-16	***
AP3B2 + ITGA10 + ITPK1	0.579	0.6		11.8057	0.0005	***
AP3B2 + ITGA10 + PTCH1	0.619	0.06		6.4765	<2e-16	***
AP3B2 + ITPK1 + PTCH1	0.630	0.05		0.2698	<2e-16	***
ITGA10 + ITPK1 + PTCH1	0.635	0.07		0.6456	<2e-16	***
AP3B2 + ITGA10 + CTNNB1	0.613	0.06		0.2395	<2e-16	***
AP3B2 + ITPK1 + CTNNB1	0.625	0.09		0.8286	<2e-16	***
AP3B2 + PTCH1 + CTNNB1	0.661	0.008	**	6.7832	<2e-16	***
ITGA10 + ITPK1 + CTNNB1	0.616	0.06		6.022	<2e-16	***
ITGA10 + PTCH1 + CTNNB1	0.669	0.006	**	6.5044	<2e-16	***
ITPK1 + PTCH1 + CTNNB1	0.672	0.007	**	0.0773	<2e-16	***
AP3B2 + ITGA10 + ITPK1 + PTCH1	0.627	0.1		5.4122	0.020	*
AP3B2 + ITGA10 + ITPK1 + CTNNB1	0.614	0.1		0.7987	<2e-16	***
AP3B2 + ITGA10 + PTCH1 + CTNNB1	0.664	0.01	*	6.4861	<2e-16	***
AP3B2 + ITPK1 + PTCH1 + CTNNB1	0.669	0.02	*	0.2227	<2e-16	***
ITGA10 + ITPK1 + PTCH1 + CTNNB1	0.672	0.01	*	0.0798	<2e-16	***
AP3B2 + ITGA10 + ITPK1 + PTCH1 + CTNNB1	0.666	0.03	*	0.2139	0.643	

Sig., significance; *, p<0.05; **, p<0.01; ***, p<0.001.

The Cox models which included *PTCH1* and *CTNNB1* like *PTCH1+CTNNB1, AP3B2+PTCH1+CTNNB1, ITGA10+PTCH1+CTNNB1*, and *ITPK1+ PTCH1+CTNNB1* achieved the best concordance and Log-rank p values (**Table 4**). Moreover, *PTCH1* and *CTNNB1* displayed the highest hazard ratios, 1.534 and 1.563 respectively in the univariate Cox Proportional Hazards with significant p- and adjusted p-values (**Table 5**). Their hazard ratios increased further in the multivariate model including *AP3B2, ITGA10, ITPK1*, *PTCH1*, and *CTNNB1* as covariates and the Schoenfeld test validated the proportional hazards assumption (**Supplementary Figure 2**). These results put forth *PTCH1* and *CTNNB1* as the markers with the highest predictive potential.

**Table 5:** Hazard Ratios for AP3B2, ITGA10, ITPK1, PTCH1, and CTNNB1 in univariate and multivariate Cox Proportional Hazards Regression Models.

Gene	Univariate Models					Multivariate Model				
	HR	Lower 95.	Upper 95.	Pr(>|z|)	Adj.p	HR	Lower 95.	Upper 95.	Pr(>|z|)	Adj.p
AP3B2	1.004	0.780	1.292	0.978	0.978	0.920	0.646	1.309	0.641	0.792
ITGA10	1.213	0.795	1.851	0.370	0.462	1.200	0.618	2.331	0.591	0.792
ITPK1	1.236	0.864	1.770	0.246	0.410	1.069	0.653	1.751	0.792	0.792
PTCH1	1.534	1.109	2.123	0.009	0.045	1.541	1.073	2.214	0.019	0.047
CTNNB1	1.563	1.074	2.273	0.019	0.047	1.667	1.106	2.512	0.014	0.047

HR, Hazards ratio; Lower 95. and Upper 95. represent lower and upper boundaries of 95% confidence interval, Adj.p: FDR adjusted p-values calculated by the “Benjamini-Hochberg” method.

Lastly, we validated the potential of *PTCH1* and *CTNNB1* as predictive biomarkers for neoadjuvant chemotherapy in ER+/HER2- breast cancer patients, by performing Cox Proportional Hazards Regression in two additional treatment arms: patients with no systemic therapy and patients who underwent endocrine therapy (**Table 6**). *PTCH1* and *CTNNB1* were associated with increased risk specifically in ER+/HER2- breast cancer patients who underwent neoadjuvant chemotherapy, while their hazard ratios were smaller than 1 and/or statistically insignificant in patients with no systemic therapy and patients who underwent endocrine therapy (**Table 6**). These findings supported that *PTCH1* and *CTNNB1* have predictive significance rather than prognostic significance. The hazard ratios for *PTCH1* and *CTNNB1* in the neoadjuvant chemotherapy arm further increased in the multivariate model suggesting an interaction between these two genes. These findings indicated that *PTCH1* and *CTNNB1* may have a high potential as predictors of resistance to taxane-based neoadjuvant chemotherapy.

**Table 6:** The hazard ratios for PTCH1, and CTNNB1 in three different arms of treatment in ER+/HER2- breast cancer patients.

Treatment Arms Gene	Univariate Models				Multivariate Model			
	HR	Lower 95.	Upper 95.	Pr(>|z|)	HR	Lower 95.	Upper 95.	Pr(>|z|)
No systemic tx (n=421)								
PTCH1	0.875	0.752	1.018	0.084	0.873	0.749	1.018	0.082
CTNNB1	0.846	0.647	1.106	0.221	0.843	0.645	1.105	0.217
*Neoadjuvant Ctx (n=361)*								
PTCH1	**1.534**	**1.109**	**2.123**	**0.009**	**1.605**	**1.133**	**2.273**	**0.007**
CTNNB1	**1.563**	**1.074**	**2.273**	**0.019**	**1.630**	**1.109**	**2.395**	**0.012**
Endocrine tx (n=1087)								
PTCH1	0.927	0.835	1.029	0.153	0.931	0.838	1.035	0.187
CTNNB1	0.779	0.654	0.928	0.005	0.782	0.657	0.932	0.005

HR: Hazards ratio, Lower 95. and Upper 95. represent lower and upper boundaries of 95% confidence interval respectively.The results for which there is a statistically significant increase in HR are written in bold.

## 4 Discussion

Several multi-gene assays have been developed over the last two decades to guide decisions on systemic therapy (12). The gene signatures in these assays were derived from mixed cohorts of breast cancer patients for prognostic purposes to estimate the risk of recurrence and distant metastasis after therapy (50). Based on the risk scores calculated with these assays, the ER+/HER2- breast cancer patients undergo only endocrine therapy in the low-risk group, or systemic chemotherapy in the high-risk group (4). However, the prognostic value of these assays does not necessarily indicate a predictive value. The discordance in the risk scores calculated with different assays and the lack of regimen-specific predictions for chemotherapy are big limitations. Additionally, breast cancer is a heterogeneous disease with variant responses to treatment in different subtypes (8, 12, 43). Distinct gene signatures may have different predictive strengths in different breast cancer subtypes. Therefore, predictors specific to distinct molecular subtypes of breast cancer are crucial. Moreover, high costs make it impossible to incorporate multi-gene assays into routine clinical practice in developing countries. These limitations were the primary motives for us to conduct this study.

In this study, we focused specifically on the ER+/HER2- breast cancer and markers specific to taxane-based chemotherapy. We analyzed multiple cohorts of ER+/HER2- breast cancer patients who underwent taxane-based neoadjuvant therapy. Our analysis revealed 18 markers of resistance to taxane-based chemotherapy, all of which are significantly upregulated in chemoresistant patients and have statistically significant positive predictive and negative predictive powers. Furthermore, we validated that the predictive strength of the 18 genes is specific to ER+/HER2- breast cancer patients.

In clinical practice, Oncotype Dx and MammaPrint are the most frequently used first-generation multi-gene assays, and EndoPredict and Prosigna are the most used second-generation multi-gene assays for ER+/HER2- breast cancer (4). The accuracy of the first-generation multi-gene assays to predict recurrence after endocrine therapy is higher in the first five years after treatment. The second-generation multi-gene assays like EndoPredict are more accurate in predicting late recurrences compared to the first-generation tests (8). Since first- and second-generation tests offer different accuracies, and each test uses a non-overlapping set of genes, we investigated the correlation of our 18-gene list with multi-gene assays of different generations. Our analysis suggested a higher correlation of the 18-gene list with Oncotype Dx in breast cancer patients.

Oncotype Dx signature is predominated by two groups of genes, one group related to hormone receptors, and another group of proliferation markers. It is now widely accepted that high expression of ER and the related genes is associated with better prognosis and sensitivity to endocrine therapy in breast cancer. On the other hand, high expression of proliferation markers such as Ki67 is associated with a worse prognosis but sensitivity to chemotherapy (4, 8). This information is the basis for the IHC4 assay of ER, PR, HER2, and Ki67. Oncotype Dx draws expression data from a set of genes highly clustered with ER and Ki67, instead of testing only these individual genes like the IHC4 assay. This may provide some robustness to Oncotype Dx (8, 51). However, our analysis in the validation set of ER+/HER2- breast cancer patients and the 1100 breast cancers in TCGA dataset demonstrated that the expressions of the ER group genes in the Oncotype Dx signature are substantially correlated with each other, and proliferation markers are strongly correlated with each other, these two clusters being negatively correlated. Therefore, in practice, the predictive advantage of Oncotype Dx over IHC4 may be limited.

The 18 gene list we identified in this study may have several advantages over multigene assays like Oncotype Dx. First, the constituents of the 18 gene list are highly independent in terms of biological functions compared to the components of the Oncotype Dx signature. Therefore, it may capture information from an extensive subset of biological processes associated with chemoresistance. Secondly, the Oncotype Dx may be more efficient in predicting resistance to endocrine therapy rather than chemotherapy, since ER-related genes constitute a big part of the signature. On the other hand, the 18 gene list we derived specifically from the data of patients who underwent taxane-based chemotherapy may be more accurate in predicting resistance to chemotherapy. Therefore, these 18 genes may have a higher predictive strength to guide the clinical decision on systemic chemotherapy in ER+/HER2- breast cancer patients. However, the size of the cohorts was a limitation to validate that. Therefore, prospective studies in larger cohorts are needed.

One other limitation of the development of predictive gene signatures is the use of pathological complete response as the surrogate of responsiveness to the therapy. Since the observation and evaluation of the pathological complete response after therapy are advantageous over the follow-up of relapse-free or overall survival, it is commonly used as the primary outcome in clinical studies to speed up the drug discovery process. However, emerging evidence suggests that its surrogacy may be different in distinct breast cancer subtypes, with questionable efficacy as a predictor of patient survival (47–49). Since publicly available datasets mostly report pathological complete response as the surrogate of therapy response and the number of patients in the datasets which included relapse-free survival or overall survival was limited, the initial steps of our algorithm for feature selection were mostly based on pathological response as the surrogate of response. This further limit the strength of the 18 genes we identified in this study. However, regarding this limitation, we further analyzed the impact of these 18 genes on relapse-free survival in another validation cohort with data on relapse-free survival.

Our KM-survival analysis of 316 ER+/HER2-patients who had undergone neoadjuvant chemotherapy validated the poor prognostic effect of *AP3B2, ITGA10, ITPK1*, and *PTCH1* as categorical variates out of 18 genes (**Figures 9A–E**). The multivariate survival models which included these four genes as dual or triple combinations were also significant mostly (**Figures 9F–O**), *AP3B2/ITPK1* gene pair achieving the lowest log-rank p-values (**Figure 9G**). These findings suggested that *AP3B2, ITGA10, ITPK1*, and *PTCH1* may be markers of resistance to taxane-based neoadjuvant chemotherapy in ER+/HER2- breast cancer patients. However, the evidence for their use as markers is insufficient yet. Therefore, a thorough investigation of both expression and mutation status together with molecular interactors of these genes should be performed to understand their role in chemoresistance in breast cancer.

The key finding of this study is the emergence of *PTCH1* and *CTNBB1* as key predictors for resistance to taxane-based neoadjuvant therapy in ER+/HER2- breast cancer. Our Cox Proportional Hazards analysis revealed that *PTCH1* and *CTNBB1* pose the highest risk for resistance, with hazard ratios over 1.5 in ER+/HER2- breast cancer patients. Their hazard ratios further increased over 1.6 in the multivariate model in the neoadjuvant therapy group. *PTCH1* and *CTNNB1* did not exhibit an increased risk in the control group and endocrine therapy group, further strengthening the predictive potential of *PTCH1* and *CTNNB1* in ER+/HER2- breast cancer. These findings together with the knowledge on the biology of these genes strongly support their predictor role in chemoresistance.

PTCH1 is a transmembrane receptor for sonic hedgehog (SHH). In the unbound form, PTCH1 captures the protein “smoothened” (SMO) which has proliferative action. The binding of SHH leads to the degradation of PTCH1, hence releasing the SMO. Then SMO dissociates Glioma-associated oncogene GLI from SUFU, activating the transcription of target genes with tumorigenic action. Due to this mechanism, PTCH1 is known as a tumor suppressor (52). However, increased expression of PTCH1 was detected in several cancers including ovarian carcinoma, lung, and prostate cancer (53, 54). More importantly, PTCH1 acts as a multidrug resistance pump, expelling chemotherapeutics like doxorubicin, and dyes like Hoesct33342, hence inducing chemoresistance (55). Since taxanes share important drug efflux pumps with doxorubicin and Hoesct33342 such as MDR1 (56), there is a high probability that taxanes can be substrates for efflux by PTCH1. Hence, *PTCH1* may be a crucial marker of resistance to taxanes and other chemotherapeutics like anthracyclines used in taxane-based chemotherapy. Moreover, targeting PTCH1 may be a key strategy to overcome taxane resistance in cancer. Accordingly, paclitaxel was shown to increase PTCH1 expression, and inhibition of proteasome suppressed PTCH1 levels and increased sensitivity of ovarian cancer cells to the paclitaxel (57). Furthermore, mutated *PTCH1* was proposed as a strong predictor of recurrence in breast cancer (58), and fusion of *PTCH1* with glioma-associated proteins was associated with oncogenic activation in different tumors (59). All these findings indicate a significant potential for PTCH1 in chemoresistance via its functions as a drug efflux pump and a hedgehog receptor.

*CTNNB1* encodes β-Catenin which is a crucial component of E-cadherin-mediated cell-cell adhesion and a downstream mediator of canonical WNT pathway. β-Catenin is significantly involved in mammary tissue development, breast cancer formation, and metastasis. Alterations in the gene expression and the localization of β-Catenin are frequently reported in breast cancer. However, the involvement of the WNT/β-Catenin pathway in breast cancer is intricate, and the expression level of β-Catenin provides incomplete information without investigation of its activity and subcellular localization (41). Therefore, there is still a discrepancy in the exact mechanisms by which WNT/β-Catenin signaling plays a role in breast cancer (60).

Similar to the controversial effects of the WNT/β-Catenin pathway reported in the literature, we observed that the signature genes that are upregulated or downregulated in the presence of constitutively active CTNNB1 were enriched in ER+/HER2- breast cancer patients with incomplete pathological response to taxane-based chemotherapy in GEO datasets (**Figures 3A, B**). However, CTNNB1 was down-regulated in ER+/HER2- breast cancer patients with incomplete pathological response to taxane-based chemotherapy in the validation cohort (**Figure 3C**). This discrepancy pointed out the necessity of investigating the activity and subcellular localization of this molecule in patient samples, besides gene expression levels, to achieve a complete understanding of the involvement of β-Catenin in chemoresistance. Despite this discrepancy in differential expression in test and validation cohorts, the high expression of *CTNNB1* was associated with decreased survival in KM-survival analysis (**Figure 3E**) and *CTNNB1* demonstrated the highest hazards ratio and significance in Cox proportional hazards regression (**Tables 4**, **5**). Therefore, our results suggested the involvement of CTNNB1 in resistance to taxane-based neoadjuvant chemotherapy in ER+/HER2- breast cancer patients.

PTCH1 and CTNNB1 take role in distinct oncogenic signaling pathways. However, our Cox Proportional Hazard models suggested an interaction between these two genes. Therefore, we searched the relevant literature to find out biological interactions between these two molecules. The non-canonical hedgehog pathway was reported to increase the expression of WNT through the involvement of PTCH1 in colon carcinoma (61). Moreover, the WNT/B-catenin pathway was reported to regulate the SHH pathway at multiple levels in different studies (62). These observations suggest that the crosstalk between SHH/PTCH1 and WNT/β-Catenin pathway may have a pivotal role in chemoresistance in ER+/HER2- breast cancer. In our prospective studies, we will dissect the mechanisms by which these pathways play a role in chemoresistance, considering the mutation status, activity, subcellular localization, and interactors of each molecule in ER+/HER2- breast cancer.

In conclusion, *PTCH1* and *CTNBB1* emerge as key markers of resistance to taxane-based neoadjuvant chemotherapy in ER+/HER2- breast cancer patients. Future studies in larger cohorts may present them as predictive markers cost-effectively incorporated into clinics to guide decisions on taxane-based chemotherapy. Detailed investigation of their molecular mechanisms may also enable the development of new molecular-targeted agents for overcoming chemoresistance in ER+/HER2- breast cancer patients. This will be addressed in our future studies.

## Data availability statement

The original contributions presented in the study are included in the article/**Supplementary Material**. Further inquiries can be directed to the corresponding author.

## Ethics statement

Ethical approval was not required for the study involving humans in accordance with the local legislation and institutional requirements. Written informed consent to participate in this study was not required from the participants or the participants’ legal guardians/next of kin in accordance with the national legislation and the institutional requirements.

## Author contributions

GO conceptualized the study, analyzed, and interpreted the genomic data to identify key markers of resistance to taxane-based neoadjuvant chemotherapy in ER+/HER2-breast cancer. GO conducted the whole study and wrote the manuscript. The author confirms being the sole contributor of this work and has approved it for publication.
