# Supplementary material for: PTCH1 and CTNNB1 emerge as pivotal predictors of resistance to neoadjuvant chemotherapy in ER+/HER2- breast cancer
Source: Front Oncol. 2023 Aug 28;13:1216438. doi: 10.3389/fonc.2023.1216438 (PMC10493393; doi:10.3389/fonc.2023.1216438)
Supplement: Supplementary file 1 [file DataSheet_1.docx]

**SUPPLEMENTARY TABLES**

**Supplementary Table 1.** Characteristics of the GEO datasets used in the study.

| **Dataset** | **Source** | **RD** | **pCR** | **PAM50 Class** | | | **Neoadjuvant Chemotherapy** |
| --- | --- | --- | --- | --- | --- | --- | --- |
|  |  |  |  | **Luminal A** | **Luminal B** | **Other** |  |
| GSE20194 | FNA | 133 | 7 | NA | NA | NA | Weekly T × 12 + FAC × 4 or  3-Weekly T × 4 + FAC × 4 |
| GSE20271 | FNA | 37 | 3 | NA | NA | NA | Weekly T × 12 + FAC × 4 |
| GSE25055 | FNA or CBX | 154 | 11 | 92 | 37 | 35 | Weekly T × 12 + FAC × 4 or  3-Weekly T × 4 + FEC × 4 or AC x 4 + T x 4 |
| GSE25065 | FNA or CBX | 94 | 19 | 49 | 31 | 33 | Weekly T × 12 + FAC × 4 or  3-Weekly T × 4 + FEC × 4 or AC x 4 + T x 4 |
| GSE32646 | CBX | 50 | 5 | NA | NA | NA | Weekly T x 12 + FEC x 4 |

AC: Adriamycin/cyclophosphamide, CBX: core biopsy, FAC: fluorouracil/adriamycin/cyclophosphamide, FEC: fluorouracil/epirubicin/cyclophosphamide, FNA: Fine-needle aspiration biopsy, NA: data not available, T: taxanes (either paclitaxel or docetaxel).

**Supplementary Table 2.** The gene ontologies enriched in the top 5 clusters of upregulated genes in chemoresistant patients.

| **Cluster 1** | **Enrichment Score: 1.614** | **Count** | **P-Value** | **Genes** |
| --- | --- | --- | --- | --- |
| GOTERM_MF | protein serine/threonine kinase activity | 7 | 0.00 | SRPK2, MAP2K4, KSR1, LIMK2, MAST2, HIPK3, CAMKK2 |
| GOTERM_MF | protein kinase activity | 6 | 0.01 | SRPK2, MAP2K4, KSR1, LIMK2, HIPK3, CAMKK2 |
| GOTERM_MF | ATP binding | 12 | 0.01 | SRPK2, MAP2K4, EHD2, AQR, KSR1, LIMK2, MAST2, ITPK1, CLCN3, HIPK3, MLH3, CAMKK2 |
| GOTERM_BP | protein phosphorylation | 6 | 0.02 | SRPK2, KSR1, LIMK2, MAST2, HIPK3, CAMKK2 |
| GOTERM_BP | positive regulation of protein phosphorylation | 4 | 0.03 | MAP2K4, LIMK2, PLA2G6, CAMKK2 |
| GOTERM_MF | protein tyrosine kinase activity | 3 | 0.05 | MAP2K4, HIPK3, CAMKK2 |
| GOTERM_BP | peptidyl-tyrosine phosphorylation | 3 | 0.07 | MAP2K4, HIPK3, CAMKK2 |
| GOTERM_BP | peptidyl-serine phosphorylation | 3 | 0.11 | SRPK2, MAST2, HIPK3 |
| **Cluster 2** | **Enrichment Score: 1.148** | **Count** | **P-Value** | **Genes** |
| GOTERM_CC | apical plasma membrane | 5 | 0.02 | CEACAM1, SLC7A8, ITPK1, CLCN3, CD44 |
| GOTERM_CC | cell surface | 5 | 0.11 | CEACAM1, CLCN3, THBS1, CD44, MPZL1 |
| GOTERM_CC | integral component of plasma membrane | 8 | 0.14 | CEACAM1, SLC7A8, ADGRE3, HFE, TNFRSF25, CLCN3, CD44, MPZL1 |
| **Cluster 3** | **Enrichment Score: 0.871** | **Count** | **P-Value** | **Genes** |
| KEGG_PATHWAY | Endocytosis | 4 | 0.11 | SH3GLB1, EHD2, SNX1, ZFYVE9 |
| GOTERM_CC | macromolecular complex | 5 | 0.14 | SH3GLB1, SNX1, KSR1, ZFYVE9, RGS11 |
| GOTERM_CC | intracellular membrane-bounded organelle | 6 | 0.15 | EHD2, SNX1, KSR1, ZFYVE9, PTCH1, CLCN3 |
| **Cluster 4** | **Enrichment Score: 0.810** | **Count** | **P-Value** | **Genes** |
| GOTERM_MF | transcriptional repressor activity, RNA polymerase II transcription regulatory region sequence-specific binding | 5 | 0.02 | ZEB1, NFX1, LRRFIP1, ZNF224, NFATC4 |
| GOTERM_BP | negative regulation of transcription from RNA polymerase II promoter | 7 | 0.08 | EID1, ZEB1, NFX1, PTCH1, LRRFIP1, ZNF224, NFATC4 |
| GOTERM_BP | regulation of transcription from RNA polymerase II promoter | 10 | 0.09 | ZNF609, ECM1, ZEB1, FOXN3, LRRFIP1, ZNF214, MED16, ZNF224, FOSL2, NFATC4 |
| GOTERM_MF | transcription factor activity, sequence-specific DNA binding | 5 | 0.10 | ZEB1, NFX1, FOXN3, FOSL2, NFATC4 |
| GOTERM_CC | chromatin | 6 | 0.19 | SRPK2, ZEB1, NFX1, FOXN3, FOSL2, NFATC4 |
| GOTERM_BP | regulation of transcription, DNA-templated | 6 | 0.20 | NFX1, ZNF747, FOXN3, LRRFIP1, ZNF214, ZNF224 |
| GOTERM_MF | RNA polymerase II transcription factor activity, sequence-specific DNA binding | 7 | 0.21 | ZEB1, NFX1, FOXN3, LRRFIP1, ZNF214, FOSL2, NFATC4 |
| GOTERM_MF | RNA polymerase II core promoter proximal region sequence-specific DNA binding | 6 | 0.33 | ZEB1, LRRFIP1, ZNF214, ZNF224, FOSL2, NFATC4 |
| GOTERM_MF | DNA binding | 6 | 0.38 | ZEB1, LRRFIP1, MAPT, RBM5, FOSL2, NFATC4 |
| GOTERM_BP | positive regulation of transcription from RNA polymerase II promoter | 5 | 0.53 | ZNF609, ZEB1, ZNF224, FOSL2, NFATC4 |
| **Cluster 5** | **Enrichment Score: 0.605** | **Count** | **P-Value** | **Genes** |
| GOTERM_CC | cell surface | 5 | 0.11 | CEACAM1, CLCN3, THBS1, CD44, MPZL1 |
| GOTERM_BP | cell migration | 3 | 0.21 | CEACAM1, THBS1, CD44 |
| GOTERM_BP | cell adhesion | 4 | 0.26 | CEACAM1, ITGA10, THBS1, CD44 |
| GOTERM_CC | extracellular exosome | 7 | 0.63 | EHD2, ECM1, CEACAM1, RPS15A, THBS1, ATP6V1D, CD44 |

GO: gene ontologies, GOTERM_CC: cellular compartments, GOTERM_MF: molecular functions, and GOTERM_BP: biological processes, KEGG_Pathway: Kyoto Encyclopedia of Genes and Genomes pathways

**Supplementary Table 3.** The 18 validated genes associated with resistance to taxane-based chemotherapy in ER+/HER2- breast cancer.

| **Symbol** | **Gene** | **Annotation** |
| --- | --- | --- |
| AP3B2 | AP-3 complex subunit beta-2 | Subunit of non-clathrin- and clathrin-associated adaptor protein complex 3 (AP-3) that plays a role in protein sorting in the late-Golgi/trans-Golgi network and/or endosomes. |
| ARL2BP | ADP-ribosylation factor-like protein 2-binding protein | Together with ARL2, plays a role in the nuclear translocation, retention, and transcriptional activity of STAT3. |
| BLOC1S1 | Biogenesis of lysosome-related organelles complex 1 subunit 1 | Component of the BLOC-1 complex, a complex that is required for normal biogenesis of lysosome-related organelles (LRO), such as platelet dense granules and melanosomes. |
| CAMKK2 | Calcium/calmodulin-dependent protein kinase kinase 2 | Calcium/calmodulin-dependent protein kinase belongs to a proposed calcium-triggered signaling cascade involved in several cellular processes. |
| ECM1 | Extracellular matrix protein 1 | Stimulates the proliferation of endothelial cells and promotes angiogenesis. Inhibits MMP9 proteolytic activity. |
| ITGA10 | Integrin alpha-10 | Integrin alpha-10/beta-1 is a receptor for collagen |
| ITPK1 | Inositol-tetrakisphosphate 1-kinase | A kinase that can phosphorylate various inositol polyphosphates such as Ins(3,4,5,6)P4 or Ins(1,3,4)P3. |
| NUDT13 | Nucleoside diphosphate-linked moiety X motif 13 | Nudix hydrolase family |
| PLA2G6 | Phospholipase A2 group VI | Catalyzes the release of fatty acids from phospholipids. |
| PTCH1 | Protein patched homolog 1 | Acts as a receptor for sonic hedgehog (SHH), indian hedgehog (IHH), and desert hedgehog (DHH). |
| RAP1GAP | Rap1 GTPase-activating protein 1 | GTPase activator for the nuclear Ras-related regulatory protein RAP-1A (KREV-1). |
| RGS11 | Regulator of G-protein signaling 11 | Inhibits signal transduction by increasing the GTPase activity of G protein alpha subunits. |
| RGS12 | Regulator of G-protein signaling 12 | Regulates G protein-coupled receptor signaling cascades. Inhibits signal transduction by increasing the GTPase activity of G protein alpha subunits. |
| RPS15A | Ribosomal protein S15a | Small subunit ribosomal protein s15a |
| SLC7A8 | Solute carrier family 7 member 8 | Sodium-independent, high-affinity transport of small and large neutral amino acids |
| ZFYVE9 | Zinc finger FYVE domain-containing protein 9 | Early endosomal protein that functions to recruit SMAD2/SMAD3 to intracellular membranes and the TGF-beta receptor. |
| ZNF214 | Zinc finger protein 214 | May be involved in transcriptional regulation. |
| ZNF609 | Zinc finger protein 609 | Transcription factor, which activates RAG1, and possibly RAG2, transcription. |

**SUPPLEMENTARY FIGURES**


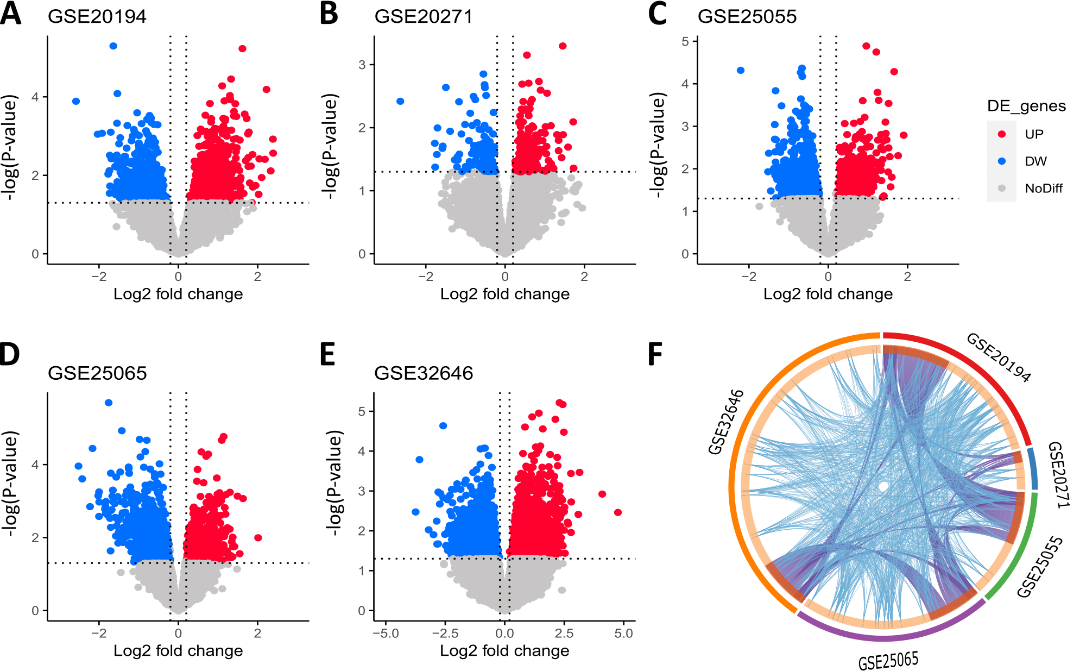


**Supplementary Figure 1.** Upregulated genes associated with resistance to taxane-based therapy in ER+/HER2- breast cancer. Volcano plots for upregulated genes in chemoresistant patients vs. chemosensitive patients in (**a**) GSE20194, (**b**) GSE20271, (**c**) GSE25055, (**d**) GSE25065, and (**e**) GSE32646 datasets. (**f**) Circos plot that shows the upregulated genes and ontologies shared by GSE20194 (red outer arc), GSE20271 (blue outer arc), GSE25055 (green outer arc), GSE25065 (purple outer arc), and GSE32646 (orange outer arc) datasets. Inner arcs represent spots for each upregulated gene in different datasets. Light orange inner arcs represent spots for genes unique to a single dataset. Dark orange inner arcs represent spots for genes shared by more than one dataset. Purple lines between the arcs link the genes that are upregulated in more than one dataset. Blue lines between the arcs link the different genes which fall into the same gene ontology term (only the ontologies where the enrichment of DEGs was statistically significant are shown in the plot).


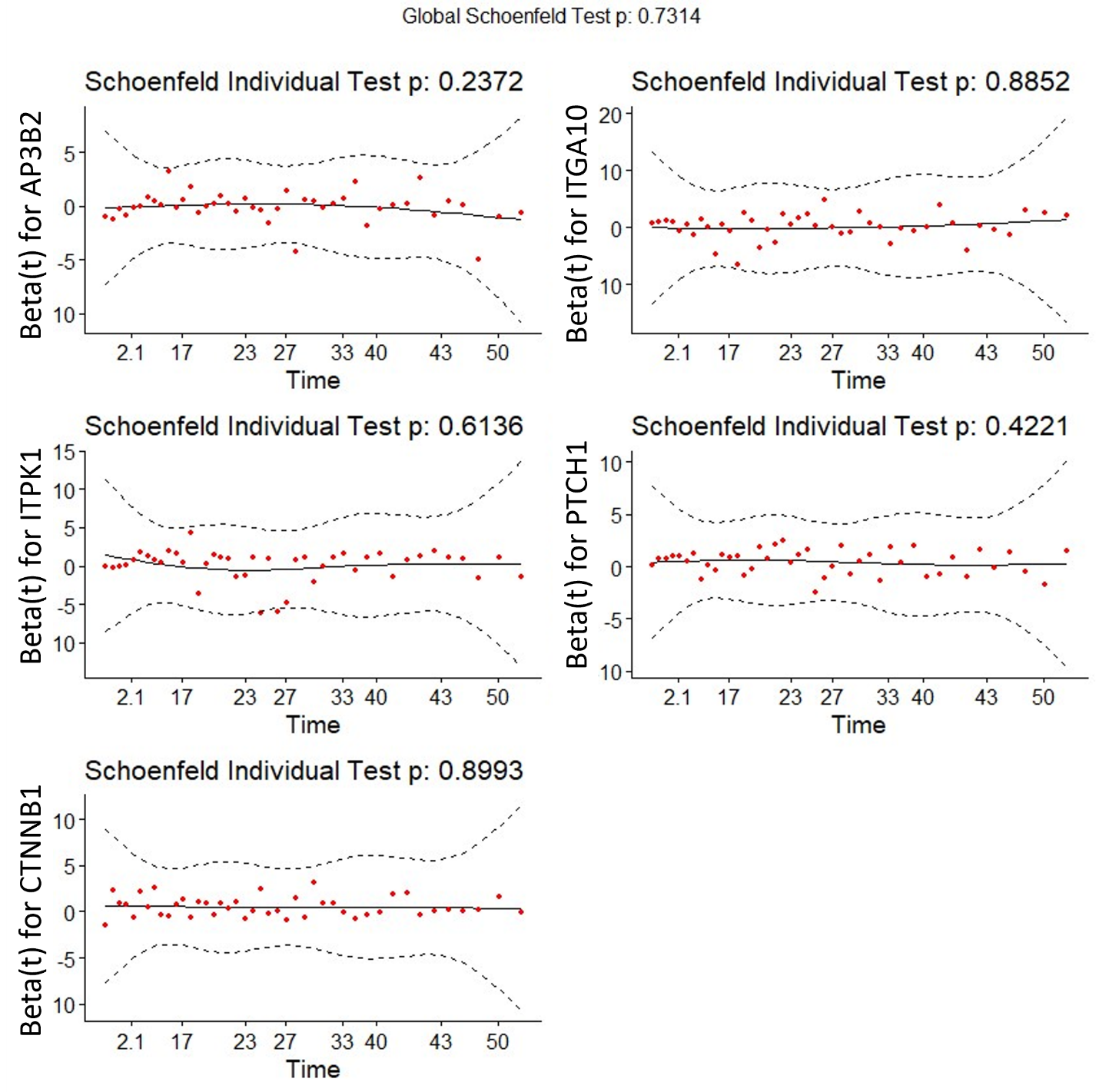


**Supplementary Figure 2.** Testing the proportional hazards assumption for AP3B2, ITGA10, ITPK1, PTCH1, and CTNNB1 in the Cox Model. The Schoenfeld test was performed to test a deviation from the proportional hazards. Since all the individual tests and the global test p-values are above 0.05, the null hypothesis was accepted indicating the proportional hazards.
